# Exploring the landscape of essential health data science skills and research challenges: a survey of stakeholders in Africa, Asia, and Latin America and the Caribbean

**DOI:** 10.3389/fpubh.2025.1523873

**Published:** 2025-03-28

**Authors:** Sally Boylan, Agklinta Kiosia, Matthew Retford, Larissa Pruner Marques, Flávia Thedim Costa Bueno, Md Saimul Islam, Anne Wozencraft

**Affiliations:** ^1^Health Data Research UK (HDR UK), HDR Global Programme, London, United Kingdom; ^2^Division of Diabetes, Endocrinology and Metabolism, Imperial College London, London, United Kingdom; ^3^Oswaldo Cruz Foundation (Fiocruz), Rio de Janeiro, Brazil; ^4^Non-Communicable Diseases, Nutrition Research Division, icddr,b, Dhaka, Bangladesh

**Keywords:** essential data science skills, data science, capacity building, global health research, low-and middle-income countries, global health challenges

## Abstract

**Background:**

Data science approaches have been pivotal in addressing public health challenges. However, there has been limited focus on identifying essential data science skills for health researchers, gaps in capacity building provision, barriers to access, and potential solutions.

**Objectives:**

This review aims to identify essential data science skills for health researchers and key stakeholders in Africa, Asia, and Latin America and the Caribbean (LAC), as well as to explore gaps and barriers in data science capacity building and share potential solutions, including any regional variations.

**Methods:**

An online survey was conducted in English, French, Spanish and Portuguese, gathering both quantitative and qualitative responses. Descriptive analysis was performed in R V4.3, and a thematic workshop approach facilitated qualitative analysis.

**Findings:**

From 262 responses from individuals across 54 low- and middle-income countries (LMICs), representing various institutions and roles, we summarised essential data science skills globally and by region. Thematic analysis revealed key gaps and barriers in capacity building, including limited training resources, lack of mentoring, challenges with data quality, infrastructure and privacy issues, and the absence of a conducive research environment.

**Conclusion and future directions:**

Respondents’ consensus on essential data science skills suggests the need for a standardised framework for capacity building, adaptable to regional contexts. Greater investment, coupled with expanded collaboration and networking, would help address gaps and barriers, fostering a robust data science ecosystem and advancing insights into global health challenges.

## 1 Introduction

“Health data science is an interdisciplinary field which is using data, methodology and tools to improve global health. It draws strength from mathematics, statistics, epidemiology and informatics to make advances in health research and outcomes” (1).

Data science approaches are increasingly used to generate insights from health research on a global scale and are integral to the World Health Organization’s (WHO) broader strategy on digital transformation (2). These insights inform policies and practices that help to address global health challenges and improve health outcomes. The importance of using effective data science approaches is underscored by the anticipated rise in the global burden of diseases, driven by factors such as population ageing, rapid urbanisation, environmental pollution and unhealthy lifestyles (3–6). Data science is re-shaping the global health landscape by providing innovative solutions, such as developing disease surveillance systems for infectious diseases, tailoring precision health interventions to local contexts, and optimising public health resource allocation through advanced data analytics (7–9, 10). As these approaches gain prominence, the demand for relevant skills to drive them forward grows exponentially. Strengthening capacity in data science is crucial to ensuring that researchers, health practitioners and other stakeholders are well-equipped to harness the potential of data science for improving health outcomes worldwide.

Several global and regional initiatives are actively working to strengthen data science capacity building in low- and middle-income countries (LMICs), aiming to enhance health research, disease surveillance, and policy-making. Key initiatives such as the Global Partnership for Sustainable Development Data (GPSDD) (11), WHO’s digital health initiatives (2), Data.org’s Capacity Accelerator Network (12) and The Global Health Network (TGHN) (13) focus on technical training, mentorship, infrastructure development and open-access resources to empower researchers and health professionals. However, despite these efforts, investment remains fragmented and long-term impact assessment mechanisms are lacking, limiting the sustained integration of data science into public health systems.

This gap is particularly concerning given the critical role of data science in addressing global health challenges, as demonstrated during the COVID-19 pandemic (37). While data-driven approaches significantly contributed to pandemic management, the broader investment in building data science capacity among health researchers in LMICs has remained inadequate (14–16). Historically, funding disparities have exacerbated this issue. At the beginning of this century, LMICs accounted for 85% of the world’s population and 92% of the global disease burden, yet received only 10% of global health research funding (17, 18). As a result, many LMICs continue to face significant gaps in health research capacity, impeding their ability to generate and apply local evidence to inform policy and improve public health (15, 19, 20).

Capacity building is defined by the United Nations as “the process of enhancing the skills, resources, and adaptive capabilities of organisations and communities, enabling them to not only cope with change, but also to thrive in a rapidly evolving environment in a sustainable manner” (21). Barriers to health research capacity building in these countries and regions include insufficient investment from governments and funders, limited coordination between researchers and policymakers, inadequate funding for executing research, and environments that are not conducive to nurturing future researchers (15, 22–24). It is essential to address these barriers by developing locally-led expertise in health data science, both to overcome the disparities in health research capacity and to address the global health challenges faced by LMICs. Local researchers, who possess a detailed understanding of their communities’ social, economic and cultural contexts, are uniquely positioned to address these challenges. Strengthening the scientific capacity of their institutions not only fosters a sustainable research culture, but also ensures that solutions are appropriately tailored and likely to be effective in the long term.

In response to these challenges, Health Data Research UK’s (HDR UK) Global programme team worked with and through The Global Health Network (TGHN) and its regional hubs, as well as global partners in Africa, Asia and Latin America and the Caribbean (LAC) to conduct this observational study, which was undertaken through an online survey that aimed to:

- identify the essential health data science skills required from those using health-related data and data science approaches in Asia, Africa and LAC
- understand and identify the current provision, gaps and requirements for data science capacity building for global health research across Asia, Africa and LAC.

The online survey was accompanied by an in-depth literature review to provide an overview of the current landscape of data science capacity building initiatives relevant to global health research in these countries and regions, while highlighting gaps (25). The overarching goal of these dual efforts was to identify the critical data science skills that can drive high-priority global health research, as well as to raise awareness, facilitate knowledge sharing, and serve as a catalyst for action and development by stakeholders in this field.

## 2 Methodology

### 2.1 Survey framework development

The development of the landscaping survey was an iterative process incorporating feedback from multiple stakeholders. This included regional partners in Africa, Asia and LAC, originally brought together through a partnership award led by The Global Health Network (TGHN) and involving HDR UK’s Global programme team. Feedback was also provided by a working group of global health researchers involved in the Global Health Data Science Hub (26). This collaboration ensured excellent representation from, and understanding of the health research ecosystems in LMICs and the three regions. During this phase, the HDR Global team evaluated various survey delivery platforms (Survey Monkey, MS Forms, Jisc), along with quantitative and qualitative analysis tools, to select the most suitable options. Key considerations in the development process included adherence to HDR UK’s Good Research Practice Guide (27), regular consultations with HDR UK’s data protection team, and iterative feedback from global partners, TGHN and the working group. The team also reviewed relevant surveys and engaged experts in crowd consensus approaches to refine the survey content. The finalised survey was piloted internally with HDR UK’s Global programme team using dummy data, followed by further piloting with global partners in Africa, Asia and LAC to ensure that the survey questions were accessible, easy to interpret and understandable in the four languages in which it was distributed: Spanish, Portuguese, French and English. Translation was carried out by data scientists who are bi-lingual, native speakers. Similarly, the survey was piloted to ensure that responses—particularly qualitative responses—could be interpreted and accurately categorised into themes. The survey was delivered online in December 2023 and January 2024 in all four languages.

Data science—and how it is used—is truly interdisciplinary and not just relevant to one kind of researcher or practitioner. In this context, a convenience sampling approach was taken; we chose not to target specific stakeholder groups, as our focus was a wider group of individual stakeholders working in health research and health services. We wanted to seek diverse views and to be as inclusive as possible, and used our global partners’ existing broad regional, country and wider networks to reach individuals across Africa, Asia and LAC working in a wide range of health research related roles and institution types.

**Figure 1:**
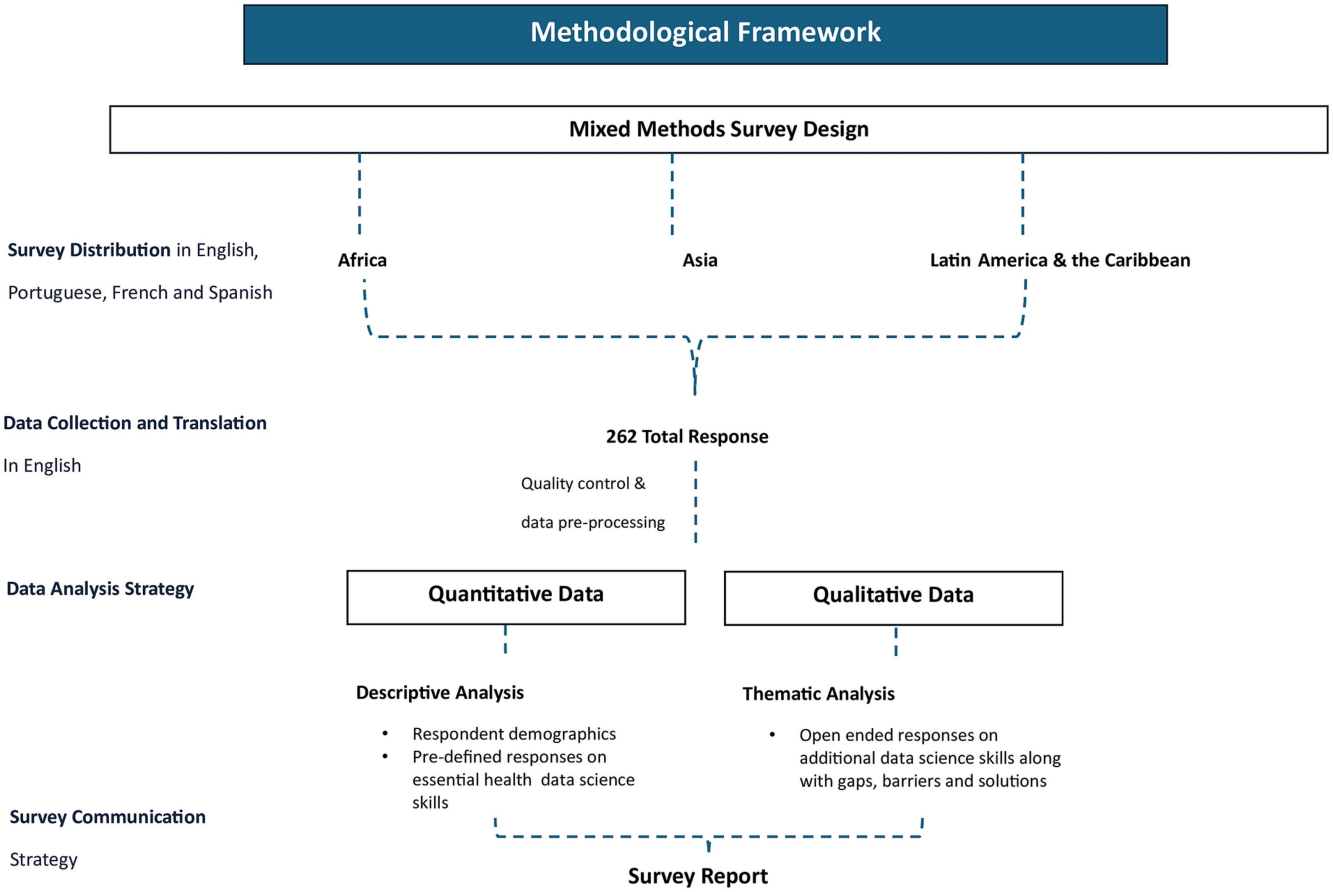
Methodology framework for landscaping survey.

The overall methodological framework is described in Figure 1.

### 2.2 Survey design

The survey consisted of three sections designed to capture comprehensive data from respondents. Respondents were required to give ‘Consent to proceed’ before beginning the survey.

#### 2.2.1 Section 1: Respondent information/profile

This section gathered details about the respondent’s role, institution type and country. Respondents selected from pre-defined drop-down menus based on expanded lists from TGHN’s ‘Essential Research Skills’ e-Delphi (28) for role and institution type, and the UN list of countries for country. Respondents were asked to respond as individuals, rather than as representatives of their country, institution or stakeholder group; and from this perspective, we sought their personal views to support this observational study.

#### 2.2.2 Section 2: Identifying essential health data science skills

**Figure 2:**
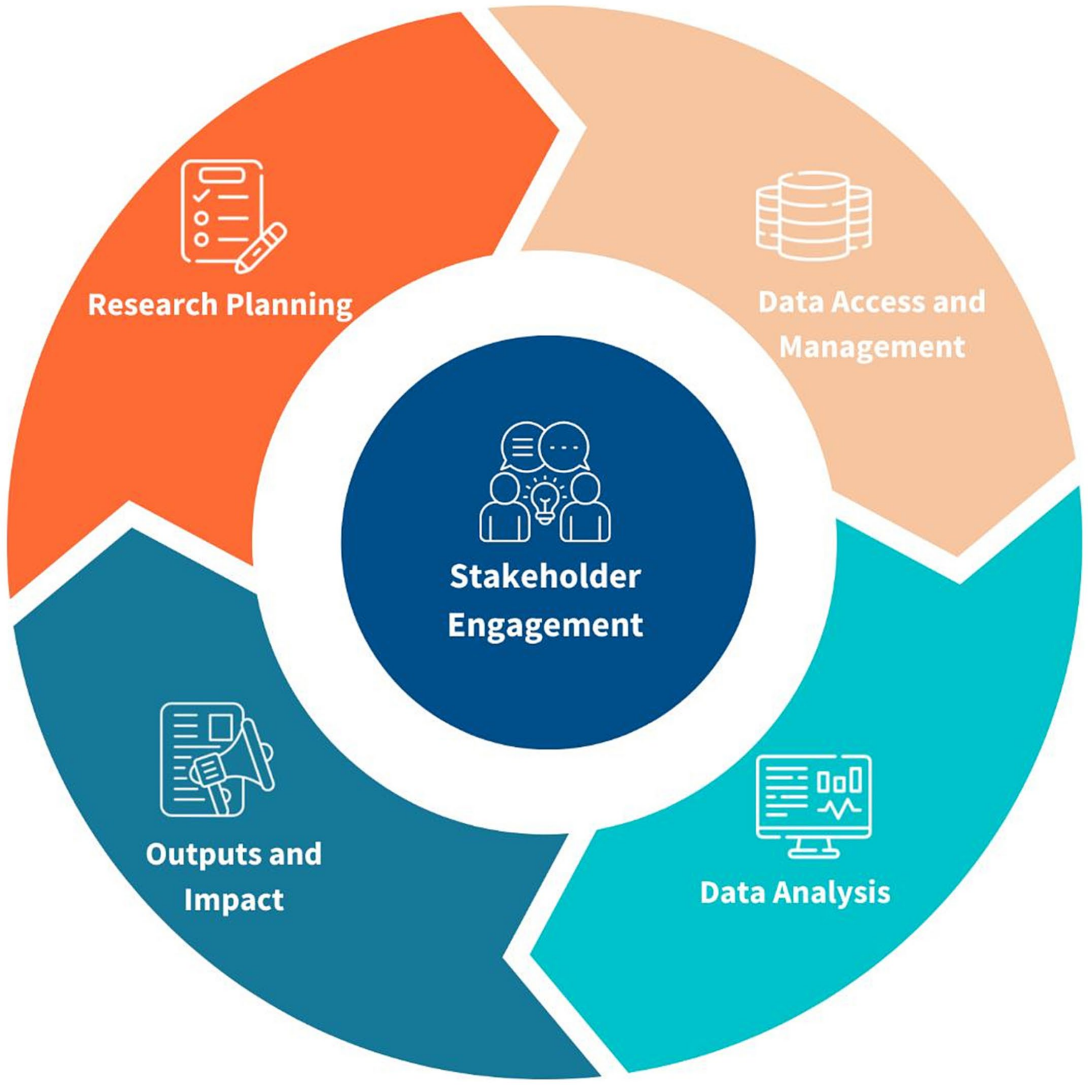
Health data research project lifecycle, developed by HDR UK’s Global programme.

Questions in this section were mandatory and respondents were asked to identify the top three essential health data science skills across the stages of the health data research project lifecycle, depicted in Figure 2. These stages included research planning, data access and management, data analysis, producing outputs and achieving impact, and community and stakeholder engagement. Skills lists for each of these stages were developed using data collected by TGHN as part of the collaboration with WHO for its report, ‘Developing an evidence-led essential research skills training curriculum’ and the health data re-use project lifecycle. Respondents could add additional skills in a free text ‘Other’ field.

#### 2.2.3 Section 3: Health data science training, gaps, barriers and solutions

This section solicited information on existing health data science training courses and resources, gaps in provision, barriers, and potential solutions. Respondents provided answers in free text fields, with responses translated into English for global analysis. The translation was verified by native speakers in HDR UK and regional partners. These questions were non-mandatory which led to variable response rates.

### 2.3 Survey dissemination strategy

The survey was distributed widely in four languages—Spanish, Portuguese, French and English—via TGHN, its global partners and HDR UK’s global networks across Africa, Asia and LAC, and remained open for 2 months, between Dec 2023–Jan 2024.

### 2.4 Data collection and storage

Data were collected through an online survey hosted on Microsoft Forms. To ensure maximum reach, the survey was widely disseminated by global partners through their regional networks as well as through the TGHN newsletter and via the Global Health Data Science digital hub (26). The team undertaking this observational study discussed ethical considerations with HDR UK’s Legal, Trust and Ethics team, and concluded that approval from an Institutional Research Board would not be required. While the survey asked for views and opinions, it did not generate any sensitive data and did not ask for provision of any data owned by institutions or institutional views, but for individual views. Ethical considerations included obtaining informed consent, ensuring anonymity and confidentiality, and securing data storage on HDR UK’s cloud platform, Box, with access restricted to HDR UK’s Global programme team and regional partners. De-identified data were prepared for analysis, with non-LMIC responses excluded and data stratified by regional groupings. To ensure validity of analysis, particularly accurate interpretation and appropriate categorisation of qualitative responses, translation was checked by data scientists based at the regional partner institutions, who are data scientists, native speakers and bi-lingual. Validation of categorisation was continuous and carried out by the team.

### 2.5 Data analysis

Data analysis followed a predefined plan to maintain consistency and validity. Quantitative data were analysed using R version 4.3.3, with descriptive statistics and visualisations employed to identify trends. Free text responses were thematically grouped through workshops using Miro, an online whiteboarding and workshop tool, and analysed at both global and regional levels. The analysis approach was tailored to the question type: drop-down menus and tick box lists allowed for quantitative analysis, while free text responses underwent qualitative analysis. For qualitative analysis, we opted to use Miro, an online collaborative tool, rather than software such as NVivo or MAXQDA. This decision was guided by the relatively small number of free-text responses, which made Miro’s visual and interactive features more suitable for our needs. Miro allowed for efficient organisation and collaborative coding in real-time, facilitating the engagement of multiple team members in the thematic grouping process. To ensure reproducibility, records of each stage of the analysis were maintained, creating a clear audit trail.

Specifically, for free text ‘Other’ responses in Section 2, a colour-coding system was implemented in Miro as a quality control measure: stickers were categorised by colour based on their relevance, with distinctions made between valid and relevant responses, irrelevant responses, and those with multiple skills that required splitting. The assessment of these stickers was conducted collaboratively by three members of HDR UK’s Global programme team. The primary goal for Section 2 was to ensure that new skills, beyond those listed in the multiple-choice options, were identified across all stages of the health data research project lifecycle, including: research planning, data access and management, data analysis, producing outputs and achieving impact, and community and stakeholder engagement. Stickers corresponding to existing essential data science skills were grouped under the relevant skills. Continuous review and validation were carried out by the analysis team, along with HDR UK’s wider Global programme team, to ensure accuracy in the thematic grouping process. Once all open-ended responses had been thematically categorised, a final review was conducted by the Global programme team to reach consensus on the newly identified skills.

For Section 3 responses (gaps, barriers, solutions), a similar approach was employed, with responses further sub-categorised by region. Key statements providing a comprehensive perspective on the challenges faced in different regions were marked for inclusion in the results section. A schematic diagram of this process is depicted in Figure 1.

## 3 Results

### 3.1 Profile of respondents

#### 3.1.1 Geographic distribution

To build a profile of the survey respondents, we asked participants to indicate their country of residence, primary job role and the type of institution they work or study at, using drop-down lists. The following sections offer detailed insights into the respondents’ represented countries, primary occupations and institutions/places of work, both globally and regionally.

**Figure 3:**
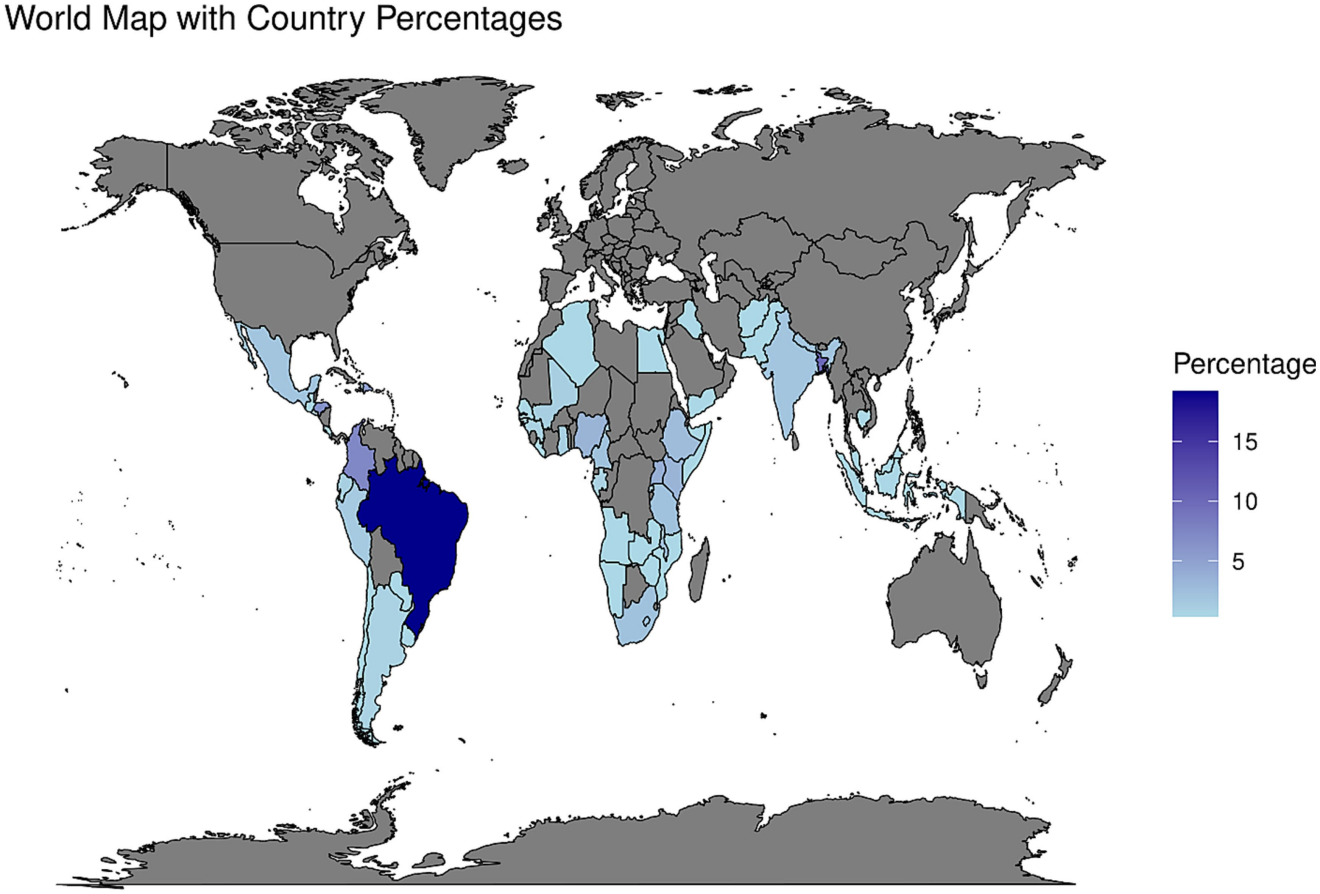
Density map depicting the percentage of survey responses received by country (*n* = 262).

The survey received responses from 276 individuals representing 61 countries worldwide. However, 14 responses were from individuals either based in high-income countries or outside the geographic focus of this survey and were excluded. In total, 262 participants from Africa, Asia and LAC, representing 54 LMICs worldwide, provided their views on the survey as shown by the density map in Figure 3.

**Figure 4:**
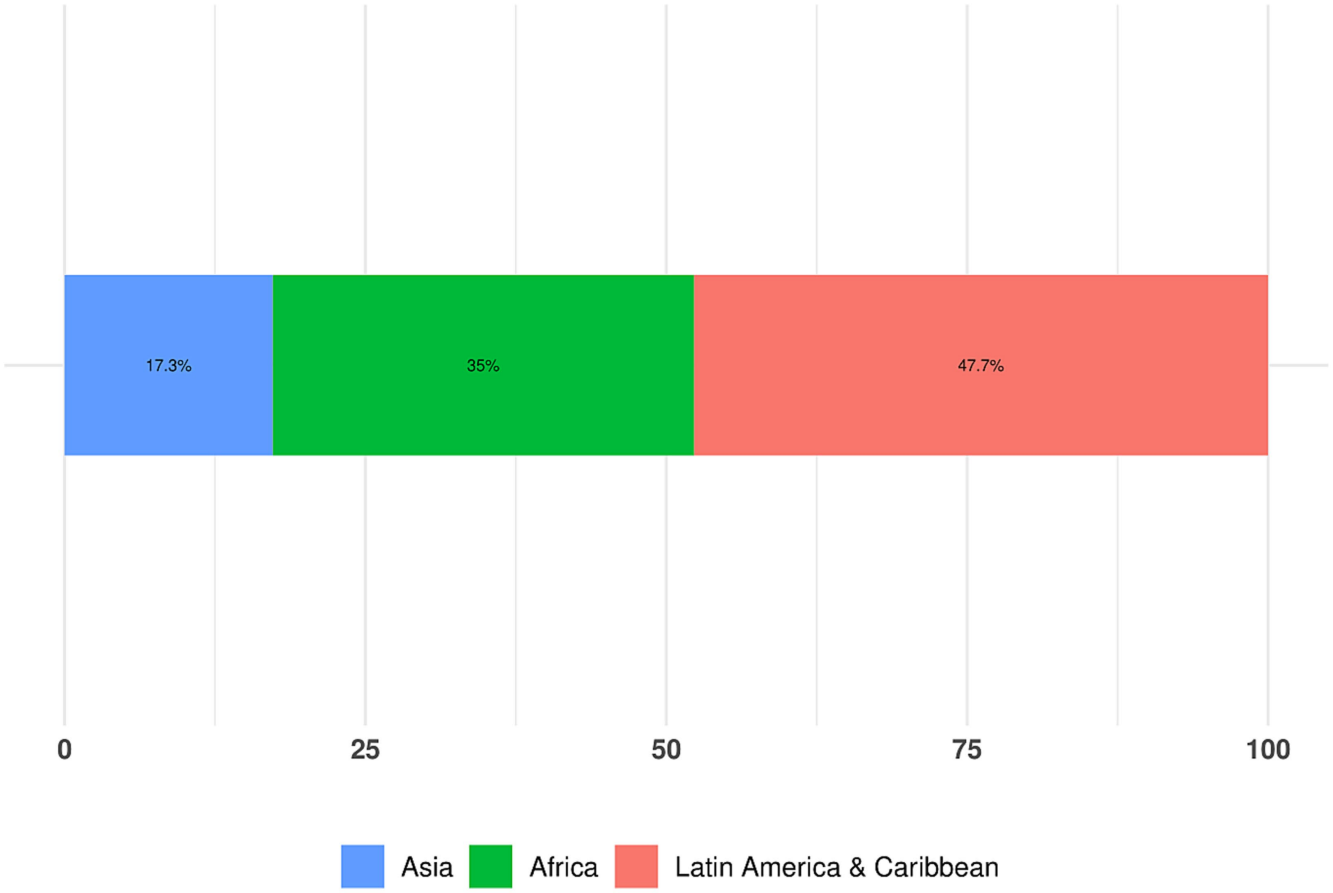
Total % survey responses received, broken down by region.

Regional representation was as follows, as also depicted in Figure 4.

- 47.7%: LAC
- 35%: Africa
- 17.3%: Asia

The majority of survey responses originated from the LAC region, representing 47.7% of total engagement. Brazil emerged as the leading country with the highest percentage of respondents (19.2%), followed by Colombia at 7.3%, Honduras at 6.5%, and Dominican Republic at 5.4%.

Africa ranked 2nd in terms of the percentage of total respondents, with African nations being the primary contributors to the survey and accounting for the majority of top 15 countries which participated in the survey.

**Figure 5:**
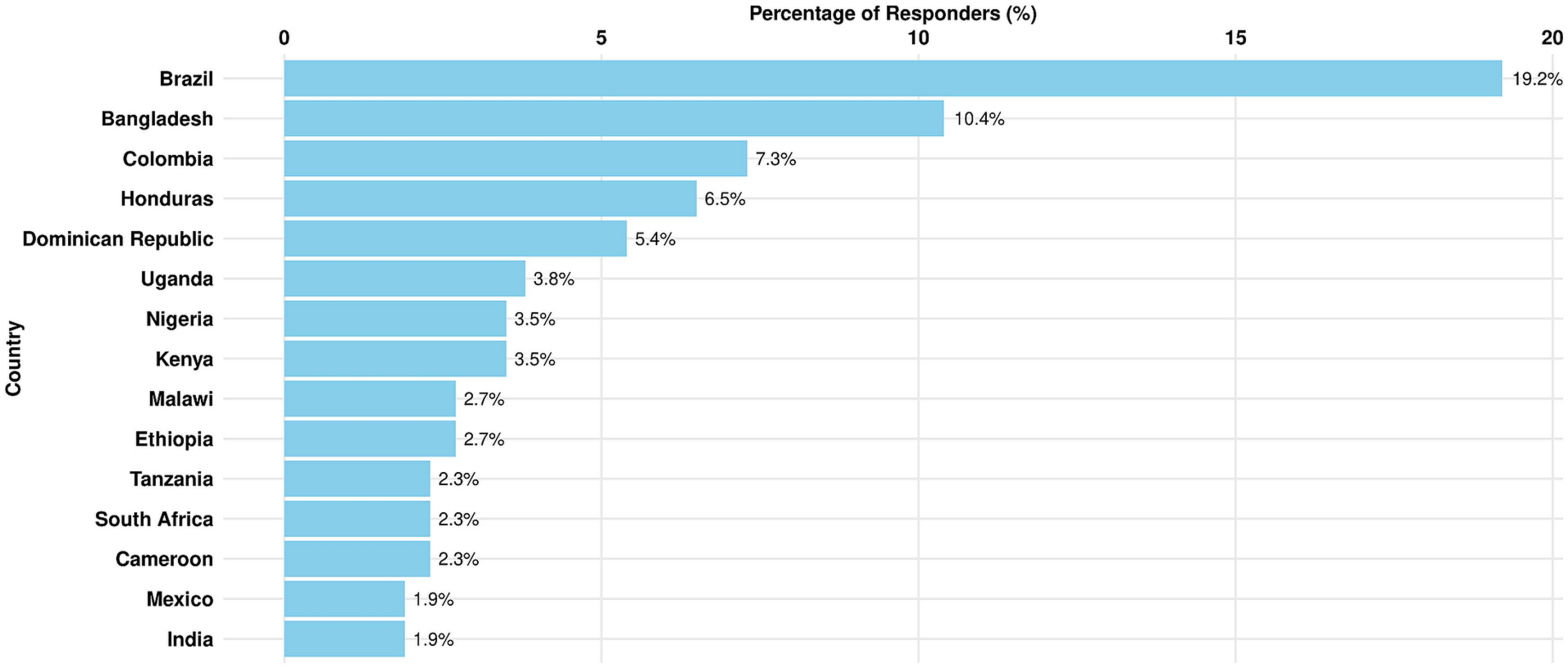
Top 15 responses in % by country—(*n*-262).

Despite Asia’s overall lower response rate compared to other regions, Bangladesh stood out as the second-highest contributor, accounting for 10.4% of responses. A detailed breakdown of the top 15 countries overall by their total percentage of respondents, is presented in Figure 5.

#### 3.1.2 Primary occupations

A wide range of primary occupations were represented across the regions, highlighting the representative nature of responses to the survey. A summary of the top seven primary occupations represented in all regions is as follows:

- 16.5%: Doctors/Clinicians
- 15.8%: Academics
- 10%: Epidemiologists
- 9.2%: Senior Researchers/Principal Investigators
- 8.5%: Data Analyst
- 8.1%: Early Career Researchers
- 6.9%: Nurse

**Figure 6:**
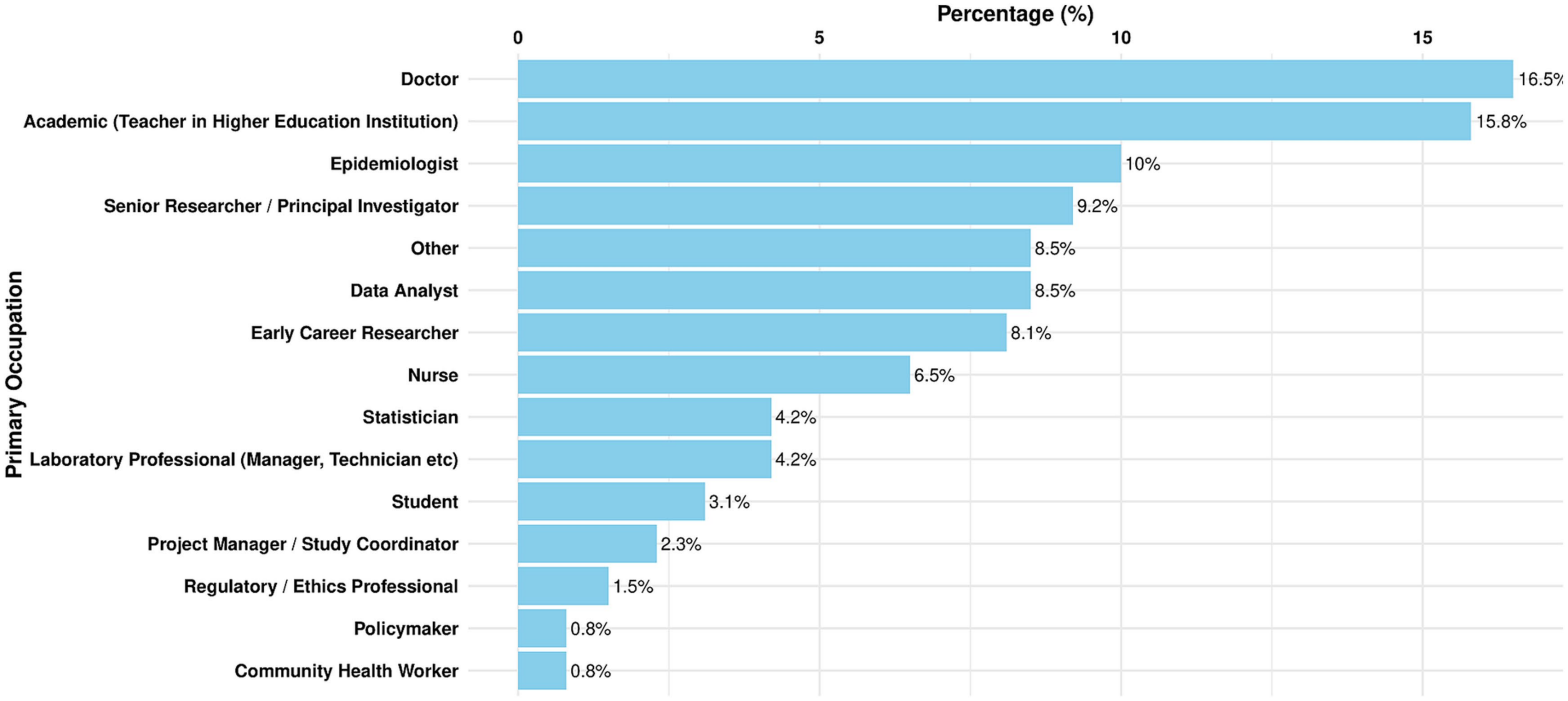
Total percentage (%) of responses in all regions, by primary occupation.

The highest percentage of responses received overall were from Doctors (Clinicians) and Academics respectively, which combined equate to just over 31% of total responses. Epidemiologist/Senior, Researcher/Principal Investigators, Data Analyst and Early Career Researcher roles were also well represented among survey participants. Combined responses from individuals in these job roles equated to just over 37%. This core 68% of responses represent roles which are critical in the field of health data science, demonstrating that the views, skills, knowledge and concerns of a range of key stakeholders were represented. The broad range of role types also reflects the multi-disciplinary nature of data science and its relevance to a broad range of job roles. Figure 6 expands on the range of respondents’ primary occupations.

It should be noted that some roles were underrepresented in survey responses, with the least represented occupations including: regulatory/ethics professionals (1.5%), policymakers (1.5%), community health workers (1.2%), funders (0.4%) and pharmacists (0.4%). This may suggest a potential limitation in survey reach.

#### 3.1.3 Institutions

The survey respondents, much like the wide variety of occupations they represented, were also affiliated with a diverse range of institutions. This is important as data science is relevant across a broad range of disciplines. It further highlights that survey responses represented perspectives from a wide range of role holders, types of institutions and geographies. A summary of the top seven institutions represented in all regions is as follows:

- 26.5%: Academic Institutions
- 16.2%: Government Scientific Research Institutes
- 14.6%: Hospitals
- 9.6%: Non-Government Organisations (NGO)
- 8.5%: International Research Organizations (IRO)
- 8.5%: Public Health Institutes
- 5.4%: Government Ministries

**Figure 7:**
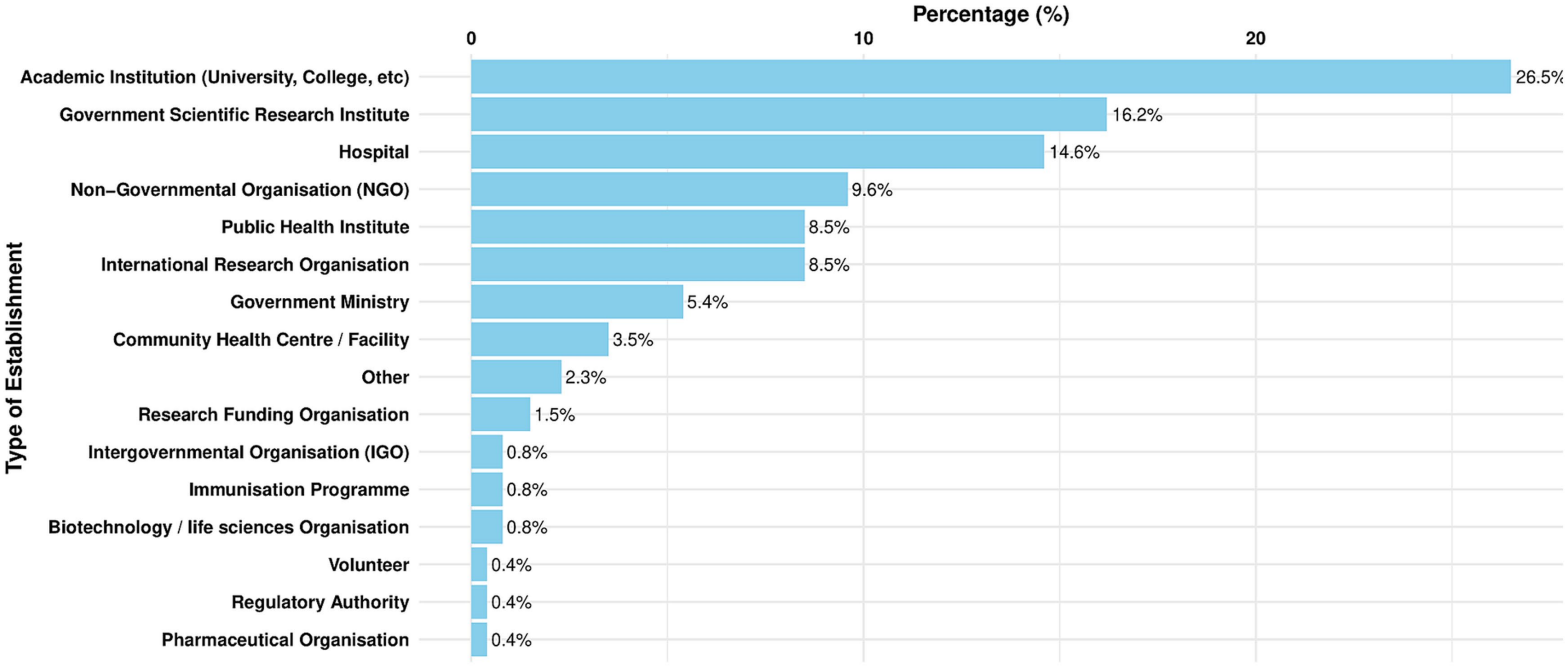
Total percentage (%) of responses from all three regions, by primary institution.

The majority of responses (26.5%), came from individuals at academic institutions, indicating a strong representation from the educational and research sectors. Government scientific research institutes followed as the next highest at 16.2%. Nearly 15% of respondents were based in hospitals, demonstrating significant input from health practitioners. NGOs accounted for almost 10% of responses, with public health institutes and international research organisations each contributing just over 8%. Although responses from regulatory authorities and pharmaceutical organisations were minimal, there was still a diverse range of establishment types represented, as illustrated in Figure 7.

### 3.2 Essential data science skills

Participants were asked to identify the top three essential health data science skills for each stage of the health data research project lifecycle (Figure 2), from a pre-defined list of skills (Supplementary Appendix 1). Participants were also invited to include additional skills beyond those listed.

While there was a high degree of consensus across all three regions on the top three essential skills for some of the stages, there was variation in the prioritisation of essential data skills across regions, underscoring the diverse regional needs and contexts.

Results of the analysis of responses for each stage of the health data research project lifecycle are shown below.

#### 3.2.1 Research planning

As outlined below, there was a high degree of consensus on the top three essential skills for Research Planning across all regions, with variation only evident in the third priority identified in the LAC region.

The top three essential skills for research planning across all regions were:

- Developing a research protocol, data science approaches and seeking ethical approval (29.4%)
- Defining the skills required in the research team and data science tools needed (22.9%)
- Understanding of research project management and evaluation (17.5%).

**Figure 8:**
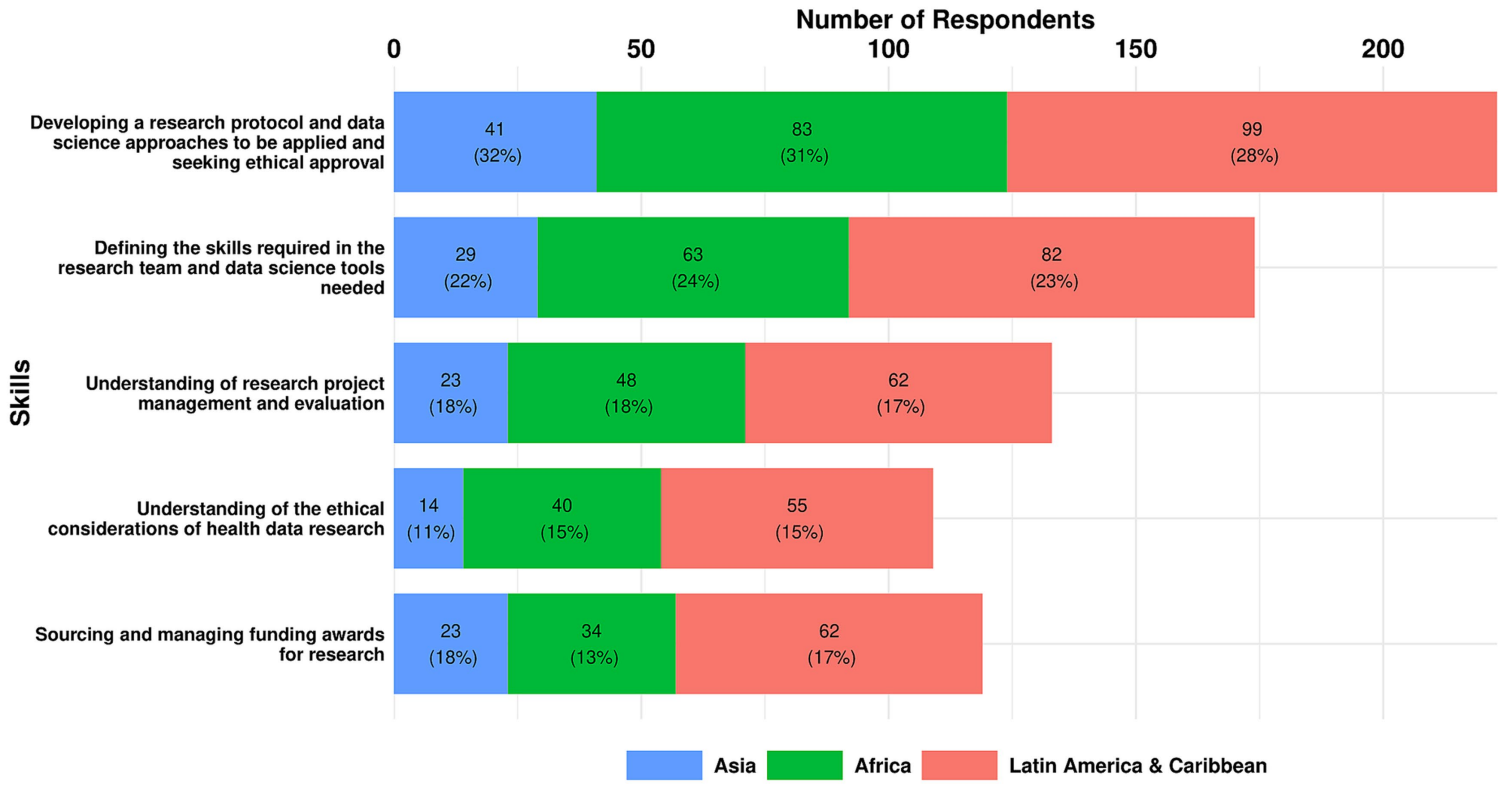
Essential research planning skills as reported by responders in each region.

A breakdown of the top three essential research planning skills by region is shown in Figure 8 and set out below.

Top three Essential Skills in Africa:

- Developing a research protocol and data science approaches, seeking ethical approval (31%)
- Defining the skills required in the research team and data science tools needed (24%)
- Understanding research project management and evaluation (18%).

Top three Essential Skills in Asia:

- Developing a research protocol and data science approaches, seeking ethical approval (32%)
- Defining the skills required in the research team and data science tools needed (22%)
- Understanding research project management and evaluation (18%).

Top three Essential Skills in LAC:

- Developing a research protocol and data science approaches, seeking ethical approval (27%)
- Defining the skills required in the research team and data science tools needed (23%)
- Sourcing and managing funding awards for research (17%).

One additional Research Planning skill was identified through analysis of all responses:

- Reviewing relevant literature and identifying research questions to be addressed using data science approaches.

#### 3.2.2 Data access and management

As set out below, there was less consensus on the top three skills for Data Access and Management, with significant variation in each region. The exception is Africa, where the top two skills mirror those identified across all three regions. This underscores the diverse priorities and contexts within each region with respect to data access and management.

The top three essential skills for data access and management across all regions were:

- Capturing and collecting data using appropriate techniques and tools (15.5%)
- Identifying health-relevant data sets for research (15%)
- Data preparation—cleaning, standardising and quality assessment of data prior to analysis (12%).

**Figure 9:**
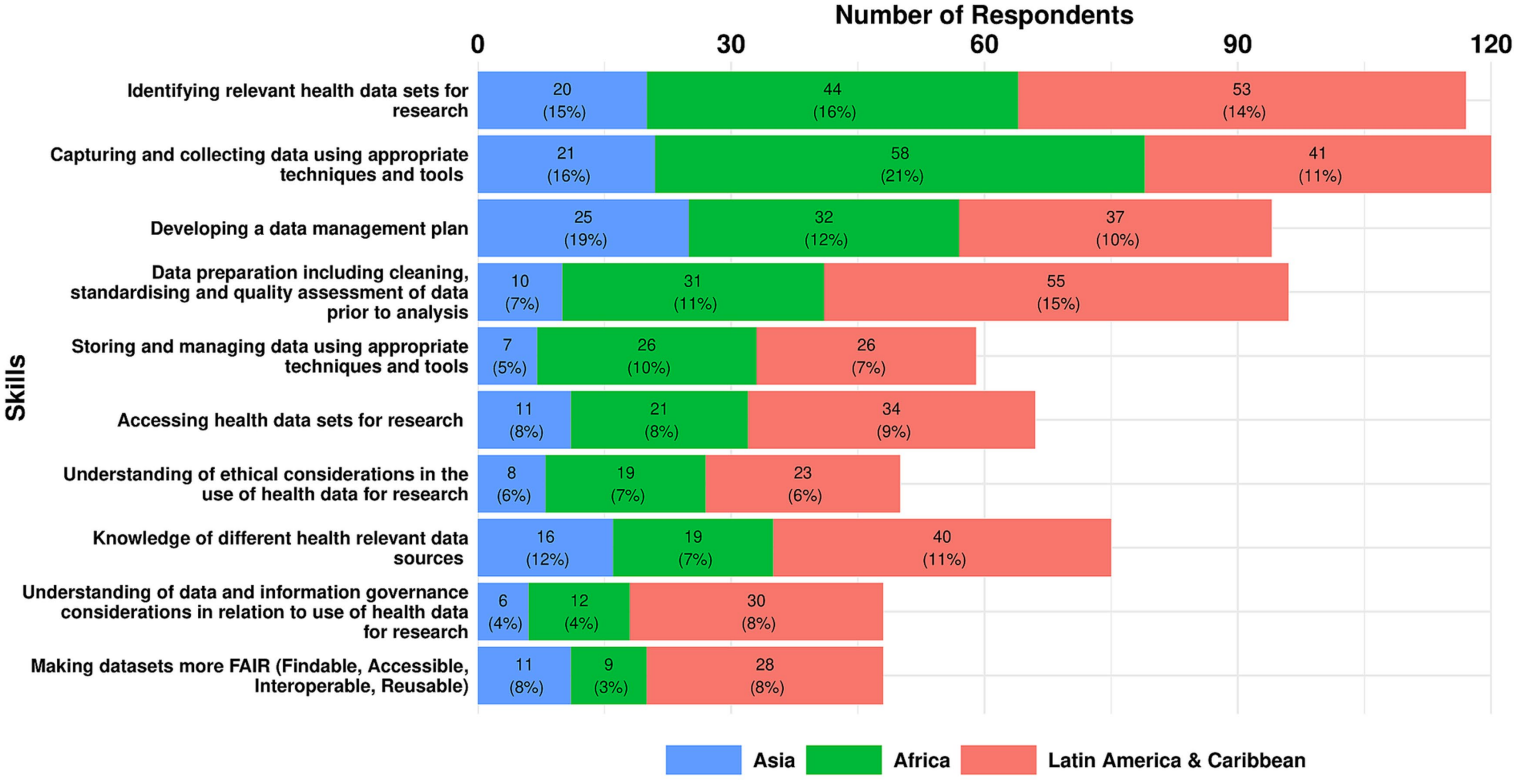
Essential data access and management skills as reported by survey respondents in each region.

A breakdown of the top three essential data access and management skills by region is shown in Figure 9 and set out below:

Top three Essential Skills in Africa:

- Capturing and collecting data using appropriate techniques and tools (21%)
- Identifying relevant health data sets for research (16%)
- Development of a data management plan (12%).

Top three Essential Skills in Asia:

- Development of a data management plan (19%)
- Capturing and collecting data using appropriate techniques and tools (16%)
- Identifying relevant health data sets for research (15%).

Top three Essential Skills in LAC:

- Data preparation—cleaning, standardising and quality assessment of data prior to analysis (15%)
- Identifying relevant health data sets for research (14%)
- Knowledge of different health relevant data sources (11%).

One additional Data Access and Management skill was identified through the analysis of all responses:

- Understanding of data security and privacy measures for managing sensitive data.

#### 3.2.3 Data analysis

The results below highlight that there was considerable consensus across the regions on the top three skills for Data Analysis, with the same skills identified by respondents in Africa and LAC (albeit with different prioritisation) as those identified across all regions. In Asia, two of the top three identified across all regions were prioritised.

The top three essential skills for data analysis across all regions were:

- Identifying appropriate statistical methods for research (25.1%)
- Analysing data using different tools and techniques (21.9%)
- Developing a data analysis plan (21.5%).

**Figure 10:**
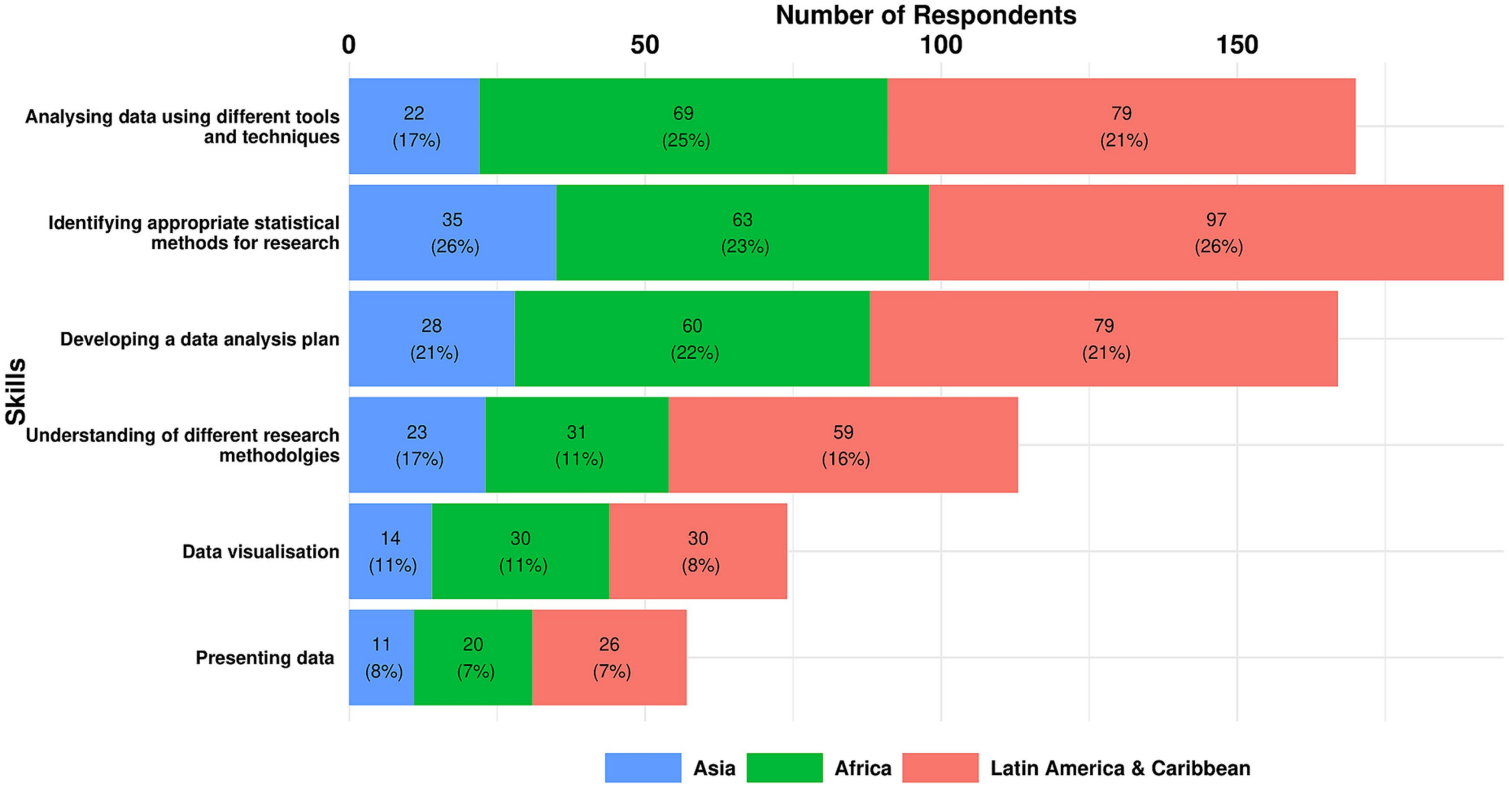
Essential data analysis skills as reported by survey respondents in each region.

A breakdown of the top three essential data analysis skills by region is shown in Figure 10 and set out below.

Top three Essential Skills in Africa:

- Analysing data using different tools and techniques (25%)
- Identifying appropriate statistical methods for research (23%)
- Developing a data analysis plan (22%).

Top three Essential Skills in Asia:

- Identifying appropriate statistical methods for research (26%)
- Developing a data analysis plan (21%)
- Understanding of different research methodologies (17%).

Top three Essential Skills in LAC:

- Identifying appropriate statistical methods for research (26%)
- Analysing data using different tools and techniques (21%)
- Developing a data analysis plan (21%).

One additional Data Analysis skill was identified through analysis of all responses:

- Understanding the research domain and clinical context for data interpretation.

#### 3.2.4 Outputs and impact

There was a high degree of consensus across all regions on the top three Outputs and Impact skills, with only Africa showing a different prioritisation. While Africa included two of the top three skills prioritised globally and by LAC and Asia, their top ranked skill was notably different and highlights the importance in the region of monitoring and evaluating the impact of research.

The top three essential skills for outputs and impact across all regions were:

- Scientific writing for journal publications (20.5%)
- Publishing and disseminating research findings through a range of mechanisms (20%)
- Developing different types of research outputs (18%).

**Figure 11:**
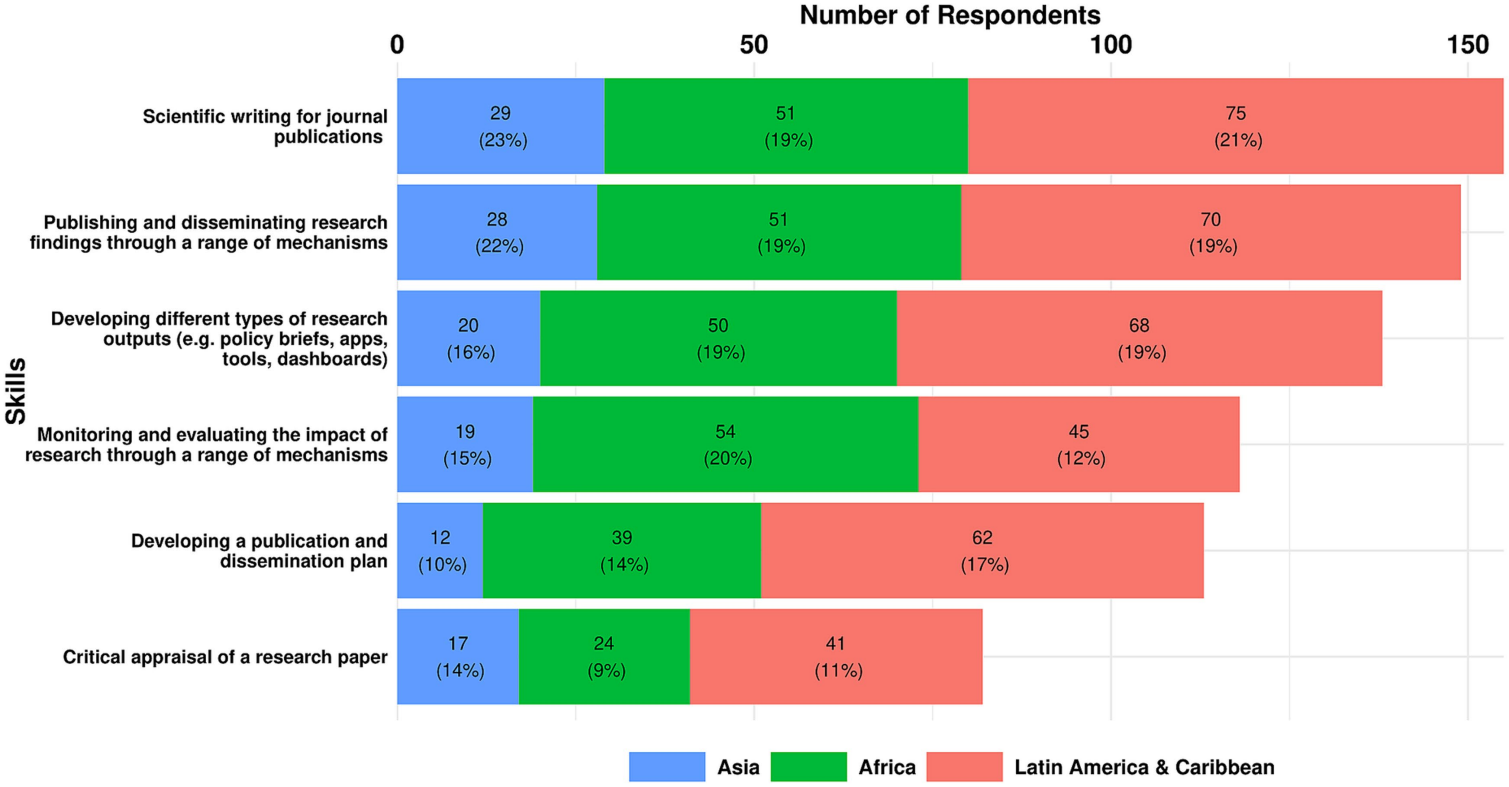
Essential outputs and impact skills identified by respondents in each region.

A breakdown of the top three essential outputs and impact skills by region is shown in Figure 11 and set out below.

Top three Essential Skills in Africa:

- Monitoring and evaluating the impact of research through a range of mechanisms (25%)
- Scientific writing for journal publications (19%)
- Publishing and disseminating research findings through a range of mechanisms (19%).

Top three Essential Skills in Asia:

- Scientific writing for journal publications (23%)
- Publishing and disseminating research findings through a range of mechanisms (22%)
- Developing different types of research outputs (16%).

Top three Essential Skills in LAC:

- Scientific writing for journal publications (21%)
- Publishing and disseminating research findings through a range of mechanisms (19%)
- Developing different types of research outputs (19%).

Three additional Outputs and Impact skills were identified through analysis of all responses:

- Understanding and use of different media and communication channels
- Adapting and translating research findings for different audiences
- Knowledge of effective methods for disseminating research insights.

#### 3.2.5 Stakeholder engagement

As shown below, there was a considerable degree of consensus on the essential Stakeholder Engagement skills. All regions agreed on the top ranked skill. Africa and LAC also agreed on the skill ranked second, but there were differing priorities in Asia and LAC for the third ranked skill, again highlighting the variation in priorities and context.

The top three essential skills for stakeholder engagement across all regions were:

- Knowledge and understanding of effective methodologies to engage with communities/stakeholders (22.9%)
- Working with different stakeholders to ensure their interests and perspectives are considered (20%)
- Communicating research evidence to influence health policy and practice (19%).

**Figure 12:**
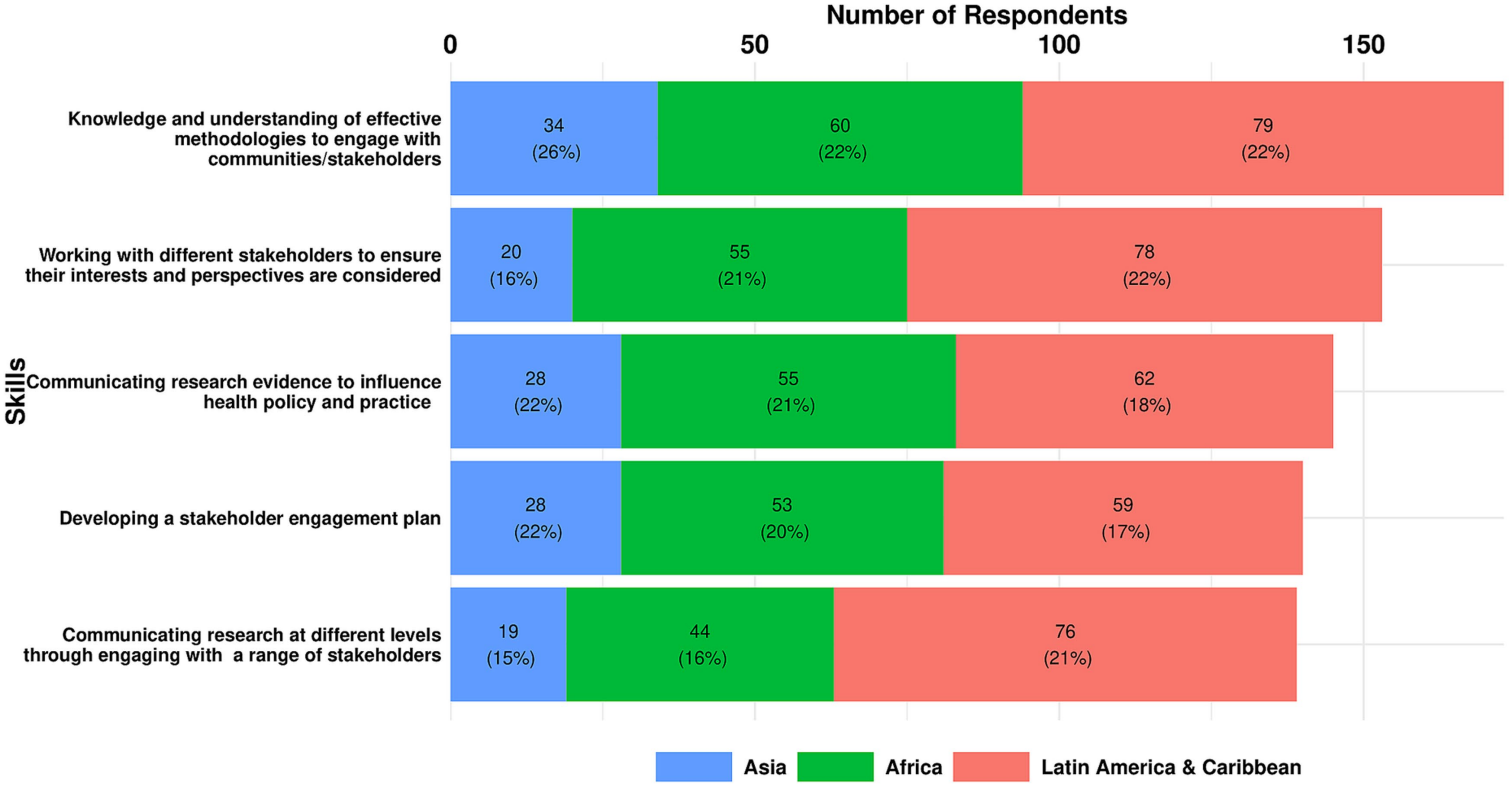
Essential stakeholder engagement skills identified by respondents in each region.

A breakdown of the top three essential stakeholder engagement skills by region is shown in Figure 12 and set out below.

Top three Essential Skills in Africa:

- Knowledge and understanding of methodologies to engage with communities/stakeholders (22%)
- Working with different stakeholders to ensure their interests and perspectives are considered (21%)
- Communicating research evidence to influence health policy and practice (21%).

Top three Essential Skills in Asia:

- Knowledge and understanding of effective methodologies to engage with communities/stakeholders (26%)
- Communicating research evidence to influence health policy and practice (22%)
- Developing a stakeholder engagement plan (22%).

Top three Essential Skills in LAC:

- Knowledge and understanding of effective methodologies to engage with communities/stakeholders (22%)
- Working with different stakeholders to ensure their interests and perspectives are considered (22%)
- Communicating research at different levels through engaging with a range of stakeholders (21%).

Three additional Stakeholder Engagement skills were identified through analysis of all responses:

- Integrating cultural understanding in research practices
- Evaluating the outcomes and impact of stakeholder engagement efforts
- Enabling stakeholders to understand and engage with research.

#### 3.2.6 Cross-cutting skills

Through analysis of the additional skills highlighted by respondents for each stage of the health data research project lifecycle, several cross-cutting or ‘soft’ skills and competencies were identified, which are applicable across the lifecycle. These are listed below.

- Communication skills and active listening
- Critical thinking and problem solving
- Teamworking, co-creation and collaboration
- Research integrity and commitment
- Willingness to adopt new technologies.

It would be important to consider these cross-cutting skills and competencies when forming a multi-disciplinary research team, to complement technical skills and facilitate effective collaboration, innovation and problem solving.

### 3.3 Gaps and barriers highlighted by respondents

To understand the current health data science capacity building landscape in each of the three regions we asked respondents to tell us about any gaps in provision of health data science capacity building initiatives and the barriers to accessing provision.

Rich and varied responses to these questions were received. While there were some differences in regional perspectives, the results revealed a number of common themes in relation to gaps and barriers including: funding challenges; lack of training and mentoring opportunities; limited opportunities for networking and collaboration; challenges with data quality, access and data infrastructure; and lack of a conducive research environment. These themes are described in more detail below.

#### 3.3.1 Funding and financing

Of all the comments received on gaps and barriers, 20% related to the theme of funding and financing. More specifically, respondents highlighted the limited funding opportunities to participate in health data science capacity building initiatives, limited funding for training and the lack of robust research funding models and mechanisms. Below are some individual perspectives shared through the survey related to this theme:

*“Lack of funding for training and relevant courses. Even if it exists, the right persons are not selected based on merit, but based on favouritism.” Statistician from Africa.*

*“Most of the people who have completed bachelor level education have basic knowledge to undertake research, but the major gap is lack of research funding, as well as regulatory bodies to ensure funding is distributed appropriately.” Academic from Asia.*

#### 3.3.2 Skills and training opportunities

Of all the comments received on gaps and barriers, 33% related to the theme of skills and training. Specific gaps and barriers highlighted related to: a lack of essential health data science skills in global health ecosystem; limited access to free and/or relevant training resources, language barriers; lack of mentoring programmes; and limited information about relevant data science capacity building opportunities for global health researchers. Below are some individual perspectives shared through the survey related to this theme:

*“Difficulty in the didactics of the courses. The language used is very technical and those who are not in the IT area have difficulty understanding and end up discouraged.” Data Analyst from LAC.*

*“Lack of professional training - even in international organizations - to develop research skills and capacity.” Early Career Researcher from Asia.*

#### 3.3.3 Data access and governance issues

Gaps and barriers were also identified for data access and governance, with 19% of all comments received relating to this theme. More specifically, respondents highlighted issues associated with: access to robust data infrastructure and connectivity; information security; data handling, including collection and management; data quality and visibility of datasets; data accessibility and sharing; and data standardisation and interoperability. Below are some individual perspectives shared through the survey related to this theme:

*“Knowledge of data handling for research and local regulations on personal data protection.” Epidemiologist from LAC.*

*“Limited access to health-related data in the country due to lack of a health database for research.” Academic from Africa.*

#### 3.3.4 Research context

Respondents also highlighted gaps and barriers within the broader global health research context, with 15% of all comments received relating to this theme. More specifically, respondents highlighted issues such as: local or regional context or environment not being conducive to quality research in institutes or teams; limited networking or collaborative opportunities; lack of access to journals or research publications; and limited institutional support. Below are some individual perspectives shared through the survey related to this theme:

*“Poor networking opportunities with those interested in developing similar skills-sets and the problems they face.” Senior Researcher from Asia.*

*“Limited access to research due to lack of internet, computers, infrastructure and technical know-how.” Nurse in Africa.*

### 3.4 Solutions proposed by respondents

In addition to highlighting gaps in and barriers to accessing capacity building opportunities, we asked respondents to tell us about any potential solutions to address the gaps and barriers identified. Many practical solutions were suggested, and these constitute a valuable source of information for all stakeholders with an interest in health research and health data science capacity building, including funders and research institutions in LMICs wishing to develop or strengthen use of data science approaches. An analysis of suggested solutions was carried out, and a number of overarching themes were identified which are described in more detail below.

#### 3.4.1 Funding and financing

Of all the suggested solutions, 12% identified the need for additional and focused funding for health data science capacity building, as well as for research more generally. More specifically, respondents highlighted issues such as the need for targeted investment in this area and for effective budgeting to directly include capacity building. Below are some individual comments shared through the survey related to this theme.

*“Service providers should be given ample funds and time to conduct action-based research.” Academic from Asia.*

*“Improvement of support to upcoming researchers in terms of the funding needed for them to attain their skills for conducting effective research.” Statistician Africa.*

#### 3.4.2 Skills and training opportunities

Of all the suggested solutions, 48% related to the theme of skills and training. More specifically, respondents suggested the following: greater promotion of training and capacity building in countries and regions; more face to face courses/workshops and distance learning courses; increased provision of free or low-cost training; easier routes to apply for training opportunities; more advanced training on specific skills; increased accessibility to data science training in multiple languages; opportunities to learn on the job; mentoring programmes. Below are some individual perspectives shared through the survey related to this theme:

*“Developing a data science mentorship hub to support junior epidemiologists or statisticians to develop their skills and marketability.” Epidemiologist from Africa.*

*“Structured training programmes with clear competencies to be developed at each stage.” Doctor in Africa.*

#### 3.4.3 Data access and governance

Of all the solutions suggested, 10% related to the theme of data access and governance. More specifically, respondents suggested the following: improvements to data infrastructure; development of more data platforms and associated tools; establishment of relevant ethics review boards; development and implementation of digital strategies; increase visibility of available datasets. Below are some individual perspectives shared through the survey related to this theme:

*“Plan and implement digital transformation in public institutes and organisations.” Data Analyst from LAC.*

*“Collaborative data sharing platforms.” Doctor in LAC.*

#### 3.4.4 Research context

Of all the suggested solutions, 7% related to the gaps and barriers identified for the theme of research context. In particular, respondents suggested the following: focus on government and/or regional initiatives; engage with communities and stakeholders to raise awareness of the importance of scientific research; provide standard policies and procedures to support data science; provided increased guidance and supportive documentation for researchers; increase partnerships and collaborations; adopt open science approaches and increase access to literature; provision of incentives; creation of an organisational culture of continuous improvement; effective team and project management; and adoption of multidisciplinary team approaches.

*“Progressively making data and results available through open science.” Senior Researcher from LAC.*

*“Have institutional work teams/data centers with capacity and time dedicated to research management.” Senior researcher from LAC.*

### 3.5 Overview of themes

An overview of the insights provided by respondents to the survey in relation to gaps in and barriers to the provision of data science capacity building, and potential solutions, is provided in Table 1.

**Table 1:** Overview of themes.

Category	Themes identified for suggested barriers and gaps	Themes identified for suggested solutions
Funding and financial	Financial issues Lack of funding for training Lack of funding or issues with funding models, or financial resources	Effective funding/budgeting mechanisms Increased investment and funding
Training and skills	Lack of training and training opportunities Knowledge gap, lack of skills and tools Lack of relevant or specific training resources Lack of resources, lack of human resources Lack of free training resources Lack of specific health data science skills Lack of mentorship Lack of mentorship	Promote training and capacity building Face to face courses/workshops Distance learning courses Free or low-cost training Effective mechanisms to apply to training opportunities More focussed/advanced training on specific skills More accessible data science training, multiple languages Resourcing and skills Opportunities to learn ‘on the job’ Mentoring programmes
Context specific	Lack of infrastructure and connectivity Context not conducive to quality research in institutes or teams Wider country or regional context issues	Community and stakeholder engagement to raise awareness / education of the importance of scientific research Country, regional or government initiatives Government adoption of interoperability tools Providing awareness training to policy makers
Knowledge and resources	Lack of material resources and support tools Lack of information and knowledge Lack of awareness of resources	Policies and procedures Resources, guidance and supportive documentation
Data access and governance	Lack of quality datasets/databases and knowledge of them Information security Lack of Access to quality data, re-use and sharing Lack of data standardisation or interoperability Data collection, handling and management Data quality	Development and implementation of digital strategies Data platforms, infrastructure and tools Increased availability of data Establishing relevant ethics review boards
Research culture and open science	Lack of incentives Lack of networking or collaborative opportunities Lack of access to journals or research publications Research culture Lack of investigation Lack of Institutional Support Limited multi-disciplinary working	Partnerships and collaborations Increase knowledge of methodologies and models Incentives Open science approaches and increased access to literature Effective team and project management Advertising and marketing, dissemination strategies Multi-disciplinary teams approaches Create an organisational culture of continuous improvement Projects and ways of working

### 3.6 Resources

As part of this landscaping survey, respondents were asked about the freely available health data science training resources or courses they were aware of in their region. Overall, respondents across the three regions identified over 80 health data science training initiatives/resources ranging from: short courses, e-Learning courses, tutorials, manuals, online capacity building communities, MOOCs, capacity building courses offered by international organisations, and formal higher education courses. These resources have been collated and made available through a ‘Resource Pack’ [(29) Global Health Data Science Resource Pack].

## 4 Discussion

The survey results underline the critical role of data science in advancing global health research and emphasise the universal recognition that data science skills are essential in accelerating health research insights across Africa, Asia and LAC. The results also demonstrate the broad applicability of the identified skills across diverse roles in public health and global health research, reinforcing the need for focused health data science capacity building initiatives that enable health researchers and practitioners to use data science approaches to accelerate the generation of impactful research insights.

### 4.1 Regional and global alignment of data science skills

Analysis of survey responses highlighted the high degree of consensus across regions on the essential health data science skills required for key stages of the health data research project lifecycle. This supports the case for the development of a structured and standardised global framework for capacity building in these skills. However, regional variations in skills prioritisation indicate the need for a common framework for health data science skills that can be tailored to regional contexts and needs.

Additionally, these findings align with existing global health initiatives, such as the WHO’s Digital Health Strategy (2), the Global Partnership for Sustainable Development Data (GPSDD) (11), and regional programmes led by The Global Health Network (TGHN) (13), which focus on capacity building through training support for data governance and infrastructure investments. Integrating recommendations from this study into these initiatives could enhance coordination and sustainability of health data science capacity building efforts.

### 4.2 Challenges and solutions for health data science capacity building

Responses to questions about the gaps in data science capacity building provided rich insights into the challenges facing health researchers and practitioners in using data science approaches to address global health priorities. A major challenge identified by respondents included the limited funding and resources for training and research. These findings align with existing research that has highlighted the chronic underfunding of health research in LMICs (30, 31). The scarcity of robust funding mechanisms inhibits the development of both essential and sustainable research infrastructure. Moreover, provision of training and related opportunities for capacity building emerged as critical areas for improvement. The survey also highlighted a lack of access to relevant training resources and mentoring opportunities, echoing findings from similar studies that document inadequate professional development and educational resources in these regions (32–34). The need for localised and accessible training solutions is evident, as current offerings often fail to address the specific needs of health data science professionals in diverse roles.

The survey findings highlighted how data access and governance issues further complicate the landscape. Challenges with data quality, infrastructure and privacy are consistent with other studies that have identified these as major barriers to effective data science practices (35, 38). Furthermore, the variability in data infrastructure across regions highlights the disparity in data management capabilities and emphasises the need for improved data governance frameworks and infrastructure investments.

In response to these challenges, a number of tangible solutions have been identified, such as increasing the number of focused data science training opportunities and resources, and improving access to these, as well as increased funding for making improvements in data quality, access and infrastructure (36). Broader solutions were also identified, including enabling increased multidisciplinary collaboration and networking, and the development of more conducive research environments.

### 4.3 A structured framework for implementation

To effectively address the identified challenges, we propose a structured framework that outlines specific actions for key stakeholders:

**Table:** 

Stakeholder	Recommended actions
Funders (e.g., government agencies, international organisations, private sector)	Increase long-term investment in health data science training programmes, provide grants for early career researchers, and establish funding streams for digital transformation, as well as approaches to improve data quality, access and infrastructure
Academic institutions	Develop standardised curricula for health data science, expand mentorship programmes, and integrate practical data science training into public health and medical education.
Governments and policy makers	Create national and regional data science hubs, support roll out of standardised health science curricula, establish data governance frameworks, and align digital health policies with global initiatives.
Research institutions and networks	Strengthen multidisciplinary collaborations, provide open access training materials, and develop knowledge-sharing platforms.

### 4.4 The need for longitudinal studies

To measure the long-term impact of capacity building initiatives, future research should incorporate longitudinal studies to track progress over time. These studies would monitor skills development and its effect on the workforce, evaluate the effectiveness of funding and policy interventions, and examine changes in data governance and infrastructure within LMICs. By providing valuable insights into the sustainability and scalability of health data science programmes, such research would help ensure continuous improvement and adaptation to evolving challenges and opportunities.

### 4.5 Study recommendations

The survey had 262 respondents representing 54 LMICs and a broad range of roles and organisations, suggesting that the results capture a wide range of perspectives and offer a comprehensive view of the current landscape. Key recommendations emerging from the survey include:

- Development of a standardised health data science curriculum framework that can be tailored for regional and country contexts
- Increased funding for focused health data science capacity building initiatives
- Development of digital strategies and increased investment at organisational, national and regional levels in digital transformation
- Creation of national/regional hubs to bring researchers together for the purposes of knowledge sharing and collaboration in health data science, with a focus on early career researchers.

## 5 Limitations

Despite the valuable insights generated from the 262 survey responses, there are a few limitations to consider.

While the survey was widely disseminated through partner networks and TGHN regional hubs, the regional distribution of responses was uneven. Nearly half (47.7%) of the responses came from Latin America and the Caribbean (LAC), compared to 35% from Africa and only 17.3% from Asia. This imbalance may introduce regional bias and limit the ability to draw specific conclusions for underrepresented areas, such as Asia, and may have affected the prioritisation of skills, gaps, barriers and solutions—especially where there may be skills, gaps, barriers and solutions associated with specific health challenges in a diverse region. However, the literature review (25) that provided a strong foundation for this study and in itself provided valuable information on gaps in each of the three regions. Additionally, there was some unevenness in the distribution of responses across different roles and types of institutions, which could further influence the representativeness of the findings. This may have led to some bias in the prioritisation of skills, gaps, barriers and solutions, given the different data science skills and competencies required by different organisations’ missions and individual role types. To address this through future studies/surveys, a targeted approach to distribution of the survey could be taken to ensure that responses are elicited from individuals across all role types and institutions, particularly those underrepresented in this study.

We acknowledge the approach taken, and the small sample size, do not allow for broad generalisations, and that findings can only be generalised to those who responded to the survey. In addition, we acknowledge the approach taken can give rise to selection bias. However, our sample was random, and we have made our observations based on the information provided by a group of respondents that represents a diverse range of roles, institution type, countries and contexts. The data also provided meaningful insights aligned with the stated objectives, even though the sample size limits the statistical power of certain analyses.

## 6 Conclusion

This survey offers critical insights into the essential data science skills required for global health research across various regions and professional contexts. It also highlights the gaps, barriers and proposed solutions necessary to address these challenges effectively. The perspectives shared by participants are intended to guide a broad range of stakeholders, including health research funders and research institutions in LMICs. To build expertise, establish clear career development pathways and ultimately improve health outcomes, it will be critical to invest in a more structured and focused approach to data science capacity building that addresses the identified gaps and barriers for global health researchers and practitioners.

Future efforts should focus on evaluating the effectiveness of the proposed solutions and frameworks in different regional contexts, with longitudinal studies assessing the impact of increased funding and tailored training programmes on data science capacity. In addition, the development of innovative data governance models and partnerships between international organisations and local institutions could provide further insights into scalable and sustainable strategies for capacity building in health data science. Overall, continued collaboration and adaptation of data science capacity building strategies to meet local needs will be essential for advancing health data science and improving global health research outcomes.

## Data Availability

The raw data supporting the conclusions of this article will be made available by the authors, without undue reservation.
