# Supplementary material for: Exploring the landscape of essential health data science skills and research challenges: a survey of stakeholders in Africa, Asia, and Latin America and the Caribbean
Source: Front Public Health. 2025 Mar 28;13:1523873. doi: 10.3389/fpubh.2025.1523873 (PMC11985845; doi:10.3389/fpubh.2025.1523873)
Supplement: Supplementary file 2 [file Table_2.DOCX]

**Table 1 – Overview of themes**

| **Category** | **Themes identified for suggested barriers and gaps** | **Themes identified for suggested solutions** |
| --- | --- | --- |
| FUNDING & FINANCIAL | - Financial issues - Lack of funding for training - Lack of funding or issues with funding models, or financial resources | - Effective funding/budgeting mechanisms - Increased investment and funding |
| TRAINING & SKILLS | - Lack of training and training opportunities - Knowledge gap, lack of skills and tools - Lack of relevant or specific training resources - Lack of resources, lack of human resources - Lack of free training resources - Lack of specific health data science skills - Lack of mentorship - Lack of mentorship | - Promote training and capacity building - Face to face courses/workshops - Distance learning courses - Free or low-cost training - Effective mechanisms to apply to training opportunities - More focussed/advanced training on specific skills - More accessible data science training, multiple languages - Resourcing and skills - Opportunities to learn 'on the job' - Mentoring programmes |
| CONTEXT SPECIFIC | - Lack of infrastructure and connectivity - Context not conducive to quality research in institutes or teams - Wider country or regional context issues | - Community and stakeholder engagement to raise awareness / education of the importance of scientific research - Country, regional or government initiatives - Government adoption of interoperability tools - Providing awareness training to policy makers |
| KNOWLEDGE & RESOURCES | - Lack of material resources and support tools - Lack of information and knowledge - Lack of awareness of resources | - Policies and procedures - Resources, guidance and supportive documentation |
| DATA ACCESS & GOVERNANCE | - Lack of quality datasets/databases and knowledge of them - Information security - Lack of Access to quality data, re-use and sharing - Lack of data standardisation or interoperability - Data collection, handling and management - Data quality | - Development and implementation of digital strategies - Data platforms, infrastructure and tools - Increased availability of data - Establishing relevant ethics review boards |
| RESEARCH CULTURE & OPEN SCIENCE | - Lack of incentives - Lack of networking or collaborative opportunities - Lack of access to journals or research publications - Research culture - Lack of investigation - Lack of Institutional Support - Limited multi-disciplinary working | - Partnerships and collaborations - Increase knowledge of methodologies and models - Incentives - Open science approaches and increased access to literature - Effective team and project management - Advertising and marketing, dissemination strategies - Multi-disciplinary teams approaches - Create an organisational culture of continuous improvement - Projects and ways of working |
