# Supplementary material for: Exploring the landscape of essential health data science skills and research challenges: a survey of stakeholders in Africa, Asia, and Latin America and the Caribbean
Source: Front Public Health. 2025 Mar 28;13:1523873. doi: 10.3389/fpubh.2025.1523873 (PMC11985845; doi:10.3389/fpubh.2025.1523873)
Supplement: Supplementary file 3 [file Table_3.DOCX]

**Supplementary Material 7 – Summary of all essential data science skills across all regions and by region**

| **Theme** | **Skil** | **All regions** | **Africa** | **Asia** | **LAC** |
| --- | --- | --- | --- | --- | --- |
| **Research Planning** |  |  |  |  |  |
|  | Developing a research protocol and data science approaches to be applied and seeking ethical approval | **29.4%** | **31%** | **32%** | **27%** |
|  | Defining the skills required in the research team and data science tools needed | **22.9%** | **24%** | **32%** | **23%** |
|  | Understanding of research project management and evaluation | **17.5%** | **18%** | **18%** | **17%** |
|  | Sourcing and managing funding awards for research | **15.7%** | **12.7%** | **17.7%** | **17.2%** |
|  | Understanding of the ethical considerations of health data research | **14.4%** | **14.9%** | **10.8%** | **15.2%** |
| **Data Access & Management** |  |  |  |  |  |
|  | Capturing and collecting data using appropriate techniques and tools | **15.5%** | **21%** | **16%** | **11%** |
|  | Identifying relevant health data sets for research | **15%** | **16%** | **19%** | **14%** |
|  | Developing a data management plan | **12.1%** | **11.8%** | **18.5%** | **10.1%** |
|  | Data preparation - cleaning, standardising and quality assessment of data prior to analysis | **12%** | **11%** | **7%** | **15%** |
|  | Knowledge of different health relevant data sources | **9.7%** | **7%** | **11.9%** | **10.9%** |
|  | Accessing health data sets for research | **8.5%** | **7.7%** | **8.1%** | **9.2%** |
|  | Storing and managing data using appropriate techniques and tools | **7.6%** | **9.6%** | **5.2%** | **7.1%** |
|  | Understanding of ethical considerations in the use of health data for research | **6.5%** | **7%** | **5.9%** | **6.2%** |
|  | Understanding of data and information governance considerations in relation to use of health data for research | **6.2%** | **4.4%** | **4.4%** | **8.2%** |
|  | Making datasets more FAIR (Findable, Accessible, Interoperable, Reusable) | **6.2%** | **3.3%** | **8.1%** | **7.6%** |
| **Data Analysis** |  |  |  |  |  |
|  | Identifying appropriate statistical methods for research | **25%** | **23%** | **26%** | **26%** |
|  | Analysing data using different tools and techniques | **22%** | **25%** | **16%** | **21%** |
|  | Developing a data analysis plan | **21.5%** | **22%** | **21%** | **21%** |
|  | Understanding of different research methodologies | **14.6%** | **11.4%** | **17.3%** | **15.9%** |
|  | Data Visualisation | **9.5%** | **11%** | **10.5%** | **8.1%** |
|  | Presenting data | **7.3%** | **7.3%** | **8.3%** | **7%** |
| **Outputs and Impact** |  |  |  |  |  |
|  | Scientific writing for journal publications | **20.5%** | **19%** | **23%** | **21%** |
|  | Publishing and disseminating research findings through a range of mechanisms | **20%** | **19%** | **22%** | **19%** |
|  | Developing different types of research outputs (e.g. policy briefs, apps, tools, dashboards) | **18%** | **19%** | **16%** | **19%** |
|  | Monitoring and evaluating the impact of research through a range of mechanisms | **15.6%** | **20.1%** | **15.1%** | **12.4%** |
|  | Developing a publication and dissemination plan | **14.9%** | **14.5%** | **9.5%** | **17.1%** |
|  | Critical appraisal of a research paper | **10.8%** | **8.9%** | **13.5%** | **11.3%** |
| **Stakeholder Engagement** |  |  |  |  |  |
|  | Knowledge and understanding of effective methodologies to engage with communities & stakeholders | **22.9%** | **22%** | **26%** | **22%** |
|  | Working with different stakeholders to ensure their interests and perspectives are considered | **20%** | **21%** | **15%** | **22%** |
|  | Communicating research evidence to influence health policy and practice | **19%** | **21%** | **22%** | **17%** |
|  | Developing a stakeholder engagement plan | **18.5%** | **19.8%** | **21.5%** | **16.5%** |
|  | Communicating research at different levels through engaging with a range of stakeholders | **18.4%** | **16.4%** | **14.6%** | **21.3%** |
