# Supplementary material for: Exploring the landscape of essential health data science skills and research challenges: a survey of stakeholders in Africa, Asia, and Latin America and the Caribbean
Source: Front Public Health. 2025 Mar 28;13:1523873. doi: 10.3389/fpubh.2025.1523873 (PMC11985845; doi:10.3389/fpubh.2025.1523873)
Supplement: Supplementary file 4 [file Data_Sheet_1.PDF]

Supplementary material 1

Landscaping Survey - Health Data Science Skills, Data Infrastructure and Data Sharing – English and French versions

English version

"Health data science is an interdisciplinary field which is using data, methodology and tools to improve global health. It draws strength from mathematics, statistics, epidemiology and informatics to make advances in health research and outcomes. Health data science helps us better understand diseases and health conditions" (London School of Hygiene and Tropical Medicine).

Development of new expertise in health data science and the skills to overcome the well-recognised barriers that limit the collecting, sharing, analysis and use of high-quality health related data, are essential to addressing the current inequalities encountered by health research teams in low-resource settings.

This landscaping survey will help identify what the current provision, gaps and requirements are for health data science capacity strengthening across Asia, Africa, Latin America and the Caribbean, and what data infrastructure and data sharing frameworks exist in each region. It will help to develop an understanding of how knowledge sharing between institutions, countries and regions is important to enabling diverse and large-scale data sharing in a trustworthy way, which can be pivotal to generating insights which improve health policy and practice and improved health outcomes for everyone.

The information you provide will be used to best enable health researchers and health practitioners in your region to use health data science approaches to improve health outcomes for everyone, and to inform policy makers and funders of the gaps and barriers to using health data science approaches.

Who are we seeking to reach with this survey?
Anyone working in global health research and data science in Africa, Asia, Latin America and the Caribbean.

About the survey
This three-part survey is being coordinated by Health Data Research Global, together with its partners, The Global Health Network, Fiocruz and icddr,b. It will take approximately 15 minutes to complete, and we greatly appreciate your time in completing it. The survey seeks to understand:

- What are the essential health data science skills required, what health data science resources/tools exist in your region, and what are the skills gaps and barriers to skills development

- What health data infrastructure and data sharing platforms, networks, governance frameworks and platforms exist in your region and what types of data you collect and use

Contact: If you have any questions about this survey, please email the HDR Global team on HDRGlobal@hdruk.ac.uk

Section 1

Personal Information

Consent to proceed

Please confirm whether you wish to proceed and complete this survey.

All data collected via this survey will be stored according to our privacy policy. Your identity will remain anonymous in all publications, release of the data and presentations of the findings. Your identity will only be requested if you give permission for us to keep in contact about this study. Your identity and contact details will not be shared beyond the research team working on this study (members of the team are from Fiocruz, TGHN Africa, icddr,b, The Global Health Network and HDR Global). For full details of HDR UK's Privacy Policy please visit https://www.hdruk.ac.uk/privacy-policy/.

Yes, I agree to processing of the data I have provided and wish to proceed and complete this survey.

2 Which of the following best describes your primary occupation?

- Academic (Teacher in Higher Education Institution)
- Community Health Worker
- Data Analyst
- Doctor
- Early Career Researcher
- Epidemiologist
- Laboratory Professional (Manager, Technician etc)
- Mathematician
- Nurse
- Pharmacist
- Policy Maker
- Project Manager/Study Coordinator
- Regulatory/Ethics Professional
- Research Funder/Grants Professional
- Senior Researcher/Principal Investigator
- Statistician
- Student
- Other*

*If you selected Other, please specify:

3 Which of the following best describes where you work/study primarily?

- Academic Institution (University, College, etc)
- Biotechnology/Life sciences organisation
- Community Health Centre/Facility
- Government Ministry
- Government Scientific Research Institute
- Hospital
- Immunisation Programme
- Intergovernmental Organisation (IGO)
- International Research Organisation
- Journal/Publishing Company
- Non-Governmental Organisation (NGO)
- Pharmaceutical Organisation
- Regulatory Authority
- Research Funding Organisation
- Technology Organisation
- Other*

*If you selected Other, please specify:

4 Which country do you work in? If you work in more than one country, please select the country in which you work primarily.

United Nations list of countries provided as a drop down list

Section 2

Essential health data science skills/competencies

We would like to understand what you consider to be the **essential health data science skills/competencies** that you would need to carry out trustworthy and effective health research using data science approaches.

Please tick the **three skills** which you consider to be most important in relation to each theme**.** Please also tell us about any **other skills** (not listed) which you consider essential in relation to the theme in the text box provided.

5 - Top 3 most essential **research planning skills**

- Developing a research protocol and data science approaches to be applied and seeking ethical approval
- Defining the skills required in the research team and data science tools needed
- Sourcing and managing funding awards for research
- Understanding of research project management and evaluation
- Understanding of the ethical considerations of health data research
- This theme is not relevant to my role

6 - Please tell us about any **other** essential health data science skills related to **Research Planning** that are missing from the list above. Please separate responses with a comma.

7 - Top 3 most essential **data access and data management skills**

- Identifying relevant health data sets for research
- Knowledge of different health relevant data sources
- Accessing health data sets for research
- Capturing and collecting data using appropriate techniques and tools
- Understanding of ethical considerations in the use of health data for research
- Developing a data management plan
- Understanding of data and information governance considerations in relation to use of health data for research
- Storing and managing data using appropriate techniques and tools
- Making datasets more FAIR (Findable, Accessible, Interoperable, Reusable)
- Data preparation including cleaning, standardising and quality assessment of data prior to analysis
- This theme is not relevant to my role

8 - Please tell us about any **other** essential health data science skills required for effective **data access and data management.** Please separate responses with a comma.

9 - Top 3 most essential **data analysis skills**

- Developing a data analysis plan
- Understanding of different research methodologies
- Identifying appropriate statistical methods for research
- Analysing data using different tools and techniques
- Presenting data
- Data visualisation
- This theme is not relevant to my role

10 - Please tell us about any **other** essential health data science skills required to carry our effective **analysis.** Please separate responses with a comma.

11 - Top 3 most essential skills for **producing outputs and achieving impact**

- Critical appraisal of a research paper
- Scientific writing for journal publications
- Developing a publication and dissemination plan
- Publishing and disseminating research findings through a range of mechanisms
- Developing different types of research outputs (e.g. policy briefs, apps, tools, dashboards)
- Monitoring and evaluating the impact of research through a range of mechanisms
- This theme is not relevant to my role

12 - Please tell us about any **other** essential health data science skills required for effectively **producing outputs and achieving impact.** Please separate responses with a comma.

13 - Top 3 most essential skills for **stakeholder engagement** (local communities, health practitioners, policy makers, health research funders)

- Developing a stakeholder engagement plan
- Knowledge and understanding of effective methodologies to engage with communities/stakeholders
- Working with different stakeholders to ensure their interests and perspectives are considered
- Communicating research evidence to influence health policy and practice
- Communicating research at different levels through engaging with a range of stakeholders
- This theme is not relevant to my role

14 - Please tell us about any **other** essential health data science skills required for effective **stakeholder engagement.** Please separate responses with a comma.

Section 3

Health data science resources, skills gaps, barriers to skills development and solutions

We are interested in learning about what freely available health data science training courses or resources are available in your region. This will help us to identify existing resources which we can signpost to and build on.

We would also like to understand any health data science knowledge or skills gaps or practical barriers to carrying out trustworthy and effective health research using data sciences approaches. and how these could be addressed.

15 - Do you know of any freely available **health data science training resources or courses** in your region?

- Yes
- No

16 - Please tell us about any freely available **health data science training courses or resources** which you are aware of in your region, and which skills they seek to develop.

(e.g. courses, toolkits or handbooks for R or other skills, 'how to' tutorials)

17 - Do you know of any **health data science skills or knowledge gaps or barriers to skills development in your region** which prevent you or others from doing more health research using data science approaches?

- Yes
- No

18 - Please tell us about **health data science knowledge and skills gaps** which prevent you from doing more health research. Please tell us about the **practical barriers that prevent you and others from doing more health research** or developing your/their own research skills and experience?

E.g. Lack of specific skills, lack of funding for training, lack of relevant courses, limited access to or knowledge of datasets, challenges in data sharing, lack of infrastructure.

19 - Do you know of any **solutions which may help to address the skills gaps and barriers to skills development**?

- Yes
- No

20 - Please tell us **how you think the skills gaps/barriers can be addressed.**

Section 4

Thank you

Thank you for participating in this survey: we greatly appreciate your time and insights. Please indicate below if you wish to receive an update on the survey findings.

21 - Would you like to receive an update on findings of this survey?

- Yes
- No

Section 5

Details

22 - Please provide your **name** for communications

23 - Please provide your **email address** for communications

French version

Enquête sur l'aménagement paysager - Compétences en Science des Données de Santé, Infrastructure des Données et Partage des Données

« La science des données de santé est un domaine interdisciplinaire qui utilise des données, des méthodes et des outils visant à améliorer la santé mondiale. Elle s'appuie sur les mathématiques, les statistiques, l'épidémiologie et l'informatique afin de réaliser des progrès dans la recherche et les résultats en matière de santé. La science des données de santé nous aide à mieux comprendre les maladies et les conditions de santé » (London School of Hygiene and Tropical Medicine).

Le développement d'une nouvelle expertise en science des données de santé et les compétences nécessaires pour surmonter les obstacles bien connus qui limitent la collecte, le partage, l'analyse et l'utilisation de données de santé de haute qualité sont essentiels pour

remédier aux inégalités actuelles rencontrées par les équipes de recherche en santé dans les environnements à faibles ressources.

Cette étude paysagère permettra d'identifier les dispositions, les lacunes et les besoins actuels en matière de renforcement des capacités dans le domaine de la science des données de santé en Asie, en Afrique, en Amérique latine et dans les Caraïbes, ainsi que les infrastructures de données et les cadres de partage de données existant dans chaque région. Elle facilitera la compréhension sur les modalités de partage des connaissances entre les institutions, les pays et les régions en vue de permettre un partage de données diversifié et à grande échelle en toute confiance, ce qui peut être essentiel pour générer des connaissances qui améliorent les politiques et les pratiques de santé et de meilleurs résultats de santé pour tout le monde.

Les informations que vous fournissez seront utilisées pour permettre aux chercheurs et aux professionnels de la santé de votre région d'utiliser au mieux les approches de la science des données de santé afin d'améliorer les résultats en matière de santé pour tous, et pour informer les décideurs politiques et les bailleurs de fonds des insuffisances et des obstacles à l'utilisation des approches de la science des données de santé.

À qui s'adresse cette enquête ?
Toute personne travaillant dans le domaine de la recherche en santé mondiale et de la science des données en Afrique, en Asie, en Amérique latine et dans les Caraïbes.

À propos de l'enquête
Cette enquête divisée en trois parties est coordonnée par Health Data Research Global, en collaboration avec ses partenaires, The Global Health Network, Fiocruz et icddr,b. Il vous faudra environ 15 minutes pour y répondre et nous vous remercions d'avoir pris le temps de le remplir. L'enquête a pour but de comprendre :

- Quelles sont les compétences essentielles requises en matière de science des données de santé, quels sont les ressources/outils de science des données de santé existant dans votre région, et quelles sont les lacunes en matière de compétences et les obstacles au développement des compétences.
- Quelles sont les infrastructures de données de santé et les plateformes de partage de données, les réseaux, les cadres de gouvernance et les plateformes qui existent dans votre région et quels sont les types de données que vous collectez et utilisez.

Contact : Si vous avez des questions sur cette enquête, veuillez envoyer un courriel à l'équipe HDR Global à l'adresse HDRGlobal@hdruk.ac.uk

Section 1
Informations personnelles
Consentement à poursuivre
Veuillez confirmer si vous souhaitez poursuivre et compléter cette enquête.

Toutes les données recueillies dans le cadre de cette enquête seront conservées conformément à notre politique de confidentialité. Votre identité restera anonyme dans toutes les publications, la diffusion des données et les présentations des résultats. Votre identité ne sera demandée que si vous nous autorisez à rester en contact avec vous au sujet de cette étude. Votre identité et vos coordonnées ne seront pas partagées en dehors de l'équipe de recherche

travaillant sur cette étude (les membres de l'équipe proviennent de Fiocruz, TGHN Africa, icddr,b, The Global Health Network et HDR Global). Pour plus de détails sur la politique de confidentialité de HDR UK, veuillez consulter https://www.hdruk.ac.uk/privacy-policy/.

Oui, j'accepte le traitement des données que j'ai fournies et je souhaite poursuivre et compléter cette enquête.

2 Laquelle des catégories suivantes décrit le mieux votre profession principale ?

- Universitaire (enseignant dans un établissement d'enseignement supérieur)
- Agent de santé communautaire
- Analyste de données
- Médecin
- Chercheur en début de carrière
- Epidémiologiste
- Professionnel de laboratoire (gestionnaire, technicien, etc.)
- Mathématicien
- Infirmier
- Pharmacien
- Décideur politique
- Gestionnaire de projet/coordonnateur d'étude
- Professionnel de la réglementation/éthique
- Professionnel du financement de la recherche/des subventions
- Chercheur principal/Investigateur principal
- Statisticien
- Étudiant
- Autre*

*Si vous avez choisi Autre, veuillez préciser :

3 Laquelle des catégories suivantes décrit le mieux l'endroit où vous travaillez/étudiez principalement ?

- Institution académique (université, collège, etc.)
- Organisation de biotechnologie/sciences de la vie
- Centre ou établissement de santé communautaire
- Ministère du gouvernement
- Institut gouvernemental de recherche scientifique
- Hôpital
- Programme de vaccination
- Organisation intergouvernementale (OIG)
- Organisation internationale de recherche
- Journal/société d'édition
- Organisation non gouvernementale (ONG)
- Organisation pharmaceutique
- Autorité de régulation
- Organisme de financement de la recherche
- Organisation technologique

- Autre*

*Si vous avez choisi Autre, veuillez préciser :

4 Dans quel pays travaillez-vous ? Si vous travaillez dans plusieurs pays, veuillez sélectionner le pays dans lequel vous travaillez principalement.

La liste des pays des Nations unies

Section 2

Compétences essentielles en science des données de santé

Nous aimerions savoir ce que vous considérez comme les **compétences essentielles en matière de science des données de santé** dont vous auriez besoin pour mener des recherches fiables et efficaces dans le domaine de la santé en utilisant les approches de la science des données.

Veuillez cocher les **trois compétences** que vous considérez comme les plus importantes pour chaque thème. Veuillez également nous indiquer **toute autre compétence** (non mentionnée) que vous considérez comme essentielle par rapport au thème dans la zone de texte prévue à cet effet.

5 - Les **3 compétences** les plus essentielles en matière de **planification de la recherche**

- Élaborer un protocole de recherche et des approches de la science des données à appliquer et obtenir une approbation éthique
- Définir les compétences requises au sein de l'équipe de recherche et les outils de science des données nécessaires
- Rechercher et gérer des financements pour la recherche
- Comprendre la gestion et de l'évaluation des projets de recherche
- Comprendre les considérations éthiques de la recherche sur les données de santé
- Ce thème n'est pas pertinent pour mon rôle

6 - Veuillez nous indiquer **toute autre** compétence essentielle en matière de science des données de santé liée à la **planification de la recherche** qui ne figure pas dans la liste ci-dessus. Veuillez séparer les réponses par une virgule.

7 - Les 3 compétences les plus essentielles en matière **d'accès aux données et de gestion des données**

- Identifier les ensembles de données sanitaires pertinentes pour la recherche
- Connaître les différentes sources de données relatives à la santé
- Accéder aux ensembles de données sur la santé pour la recherche
- Saisir et collecter des données à l'aide de techniques et d'outils appropriés
- Comprendre les considérations éthiques dans l'utilisation des données de santé pour la recherche
- Élaborer un plan de gestion des données

- Comprendre les considérations relatives à la gouvernance des données et de l'information dans le cadre de l'utilisation des données relatives à la santé à des fins de recherche
- Stocker et traiter les données à l'aide de techniques et d'outils appropriés
- Rendre les ensembles de données plus FAIR (trouvables, accessibles, interopérables, réutilisables)
- Préparer des données, dont le nettoyage, la normalisation et l'évaluation de la qualité des données avant l'analyse.
- Ce thème n'est pas pertinent pour mon rôle

8 - Veuillez nous indiquer **toute autre** compétence essentielle en matière de science des données de santé requise pour un **accès aux données et une gestion des données** efficaces. Veuillez séparer les réponses par une virgule.

9 - Les **3 compétences** les plus essentielles en matière **d'analyse de données**
- Élaborer un plan d'analyse des données
- Comprendre les différentes méthodologies de recherche
- Identifier les méthodes statistiques appropriées pour la recherche
- Analyser les données à l'aide de différents outils et techniques
- Présenter les données
- Visualiser les données
- Ce thème n'est pas pertinent pour mon rôle

10 - Veuillez nous indiquer **toute autre** compétence essentielle en matière de science des données de santé requise pour réaliser une **analyse efficace.** Veuillez séparer les réponses par une virgule.

11 - Les 3 compétences les plus essentielles pour **produire des résultats et avoir un impact**
- Évaluation critique d'un document de recherche
- Rédaction scientifique pour les publications dans les revues
- Élaboration d'un plan de publication et de diffusion
- Publication et diffusion des résultats de la recherche par le biais d'une série de mécanismes
- Élaborer différents types de résultats de recherche (par exemple, des notes d'orientation, des applications, des outils, des tableaux de bord)
- Contrôler et évaluer l'impact de la recherche par le biais d'une série de mécanismes.
- Ce thème n'est pas pertinent pour mon rôle

12 - Veuillez nous indiquer **toute autre** compétence essentielle en matière de science des données de santé requise pour **produire efficacement des résultats et avoir un impact**. Veuillez séparer les réponses par une virgule.

13 - Les 3 compétences les plus essentielles pour **l'engagement des parties prenantes** (communautés locales, praticiens de la santé, décideurs politiques, financeurs de la recherche en santé)

- Élaboration d'un plan d'engagement des parties prenantes
- Connaissance et compréhension des méthodologies efficaces pour s'engager avec les communautés/parties prenantes
- Collaboration avec différentes parties prenantes pour s'assurer que leurs intérêts et leurs points de vue sont pris en compte
- Communication des résultats de la recherche pour influencer les politiques et les pratiques en matière de santé
- Communication de la recherche à différents niveaux en s'engageant auprès d'une série de parties prenantes.
- Ce thème n'est pas pertinent pour mon rôle

14 - Veuillez nous indiquer **toute autre** compétence essentielle en matière de science des données de santé requise pour un **engagement efficace des parties prenantes**. Veuillez séparer les réponses par une virgule.

Section 3

Ressources en science des données de santé, lacunes en matière de compétences, obstacles au développement des compétences et solutions

Nous souhaitons connaître les cours de formation ou les ressources en science des données de santé disponibles gratuitement dans votre région. Cela nous aidera à identifier les ressources existantes vers lesquelles nous pourrons renvoyer et sur lesquelles nous pourrons nous appuyer.

Nous aimerions également comprendre les lacunes en matière de connaissances ou de compétences dans le domaine de la science des données de santé, ainsi que les obstacles pratiques à la réalisation d'une recherche en santé fiable et efficace à l'aide d'approches fondées sur la science des données, et la manière dont ces lacunes ou obstacles pourraient être comblés.

15 - Connaissez-vous des **ressources ou des cours de formation en science des données de santé** disponibles gratuitement dans votre région ?

- Oui
- Non

16 - Veuillez nous indiquer **les cours de formation ou les ressources en science des données de santé** disponibles gratuitement dans votre région, et les compétences qu'ils cherchent à développer
(Par exemple, des cours, des boîtes à outils ou des manuels pour R ou d'autres compétences, tutoriels "comment faire").

17 - Connaissez-vous des **insufficiances en matière de compétences ou de connaissances en science des données de santé ou des obstacles au développement des compétences** dans votre région qui vous empêchent ou empêchent d'autres personnes de faire plus de recherche en santé en utilisant des approches de science des données ?

- Oui
- Non

18 - Veuillez nous indiquer les lacunes en matière de connaissances et de compétences dans le domaine **de la science des données de santé** qui vous empêchent de mener davantage de recherches dans le domaine de la santé. Veuillez nous indiquer les **obstacles pratiques qui vous empêchent, vous et d'autres personnes, de mener davantage de recherches dans le domaine de la santé** ou de développer vos compétences et votre expérience en matière de recherche ?

Par exemple, le manque de compétences spécifiques, le manque de financement pour la formation, le manque de cours pertinents, l'accès limité aux ensembles de données ou la connaissance limitée de ceux-ci, les défis liés au partage des données, le manque d'infrastructures.

19 - Connaissez-vous **des solutions qui pourraient contribuer à combler les insuffisances en matière de compétences et à lever les obstacles au développement des compétences ?**

- Oui
- Non

20 - Veuillez nous dire **comment vous pensez que les lacunes/obstacles en matière de compétences peuvent être résolus.**

Section 4
Merci pour votre aide.

Nous vous remercions d'avoir participé à cette enquête : nous apprécions grandement le temps que vous nous avez consacré et les informations que vous nous avez fournies. Veuillez indiquer ci-dessous si vous souhaitez recevoir une mise à jour des résultats de l'enquête.

21 - Souhaitez-vous recevoir une mise à jour des résultats de cette enquête ?

- Oui
- Non

Section 5

Détails

22 - Veuillez indiquer votre **nom** pour les communications

23 - Veuillez indiquer votre **adresse électronique** pour les communications
