# Supplementary material for: Exploring the landscape of essential health data science skills and research challenges: a survey of stakeholders in Africa, Asia, and Latin America and the Caribbean
Source: Front Public Health. 2025 Mar 28;13:1523873. doi: 10.3389/fpubh.2025.1523873 (PMC11985845; doi:10.3389/fpubh.2025.1523873)
Supplement: Supplementary file 5 [file Data_Sheet_2.PDF]

Supplementary material 2

Landscaping Survey - Health Data Science Skills, Data Infrastructure and Data Sharing – Portuguese and Spanish versions

English	Portuguese	Spanish
**Landscaping Survey - Health Data Science Skills, Data Infrastructure and Data Sharing**<br><br>"Health data science is an interdisciplinary field which is using data, methodology and tools to improve global health. It draws strength from mathematics, statistics, epidemiology and informatics to make advances in health research and outcomes. Health data science helps us better understand diseases and health conditions" (London School of Hygiene and Tropical Medicine).<br><br>Development of new expertise in health data science and the skills to overcome the well-recognised barriers that limit the collecting, sharing, analysis and use of high-quality health related data, are essential to addressing the current inequalities encountered by health research teams in low-resource settings.<br><br>This landscaping survey will help identify what the current provision, gaps and requirements are for health data science capacity strengthening across Asia, Africa, Latin America and the Caribbean, and what data infrastructure and data sharing frameworks exist in each region. It will help to develop an understanding of how knowledge sharing between institutions,	**Questionário - Habilidades em Ciência de Dados em Saúde, Infraestrutura de Dados e Compartilhamento de Dados**<br><br>"Ciência de dados em saúde é um campo interdisciplinar que utiliza dados, metodologia e ferramentas para melhorar a saúde global. Ele se beneficia da matemática, estatística, epidemiologia e informática para avançar na pesquisa em saúde e seus resultados. A ciência de dados em saúde nos ajuda a compreender melhor doenças e condições de saúde" (London School of Hygiene and Tropical Medicine).<br><br>O desenvolvimento de novas competências em ciência de dados em saúde e as habilidades para superar as barreiras bem reconhecidas que limitam a coleta, compartilhamento, análise e uso de dados de saúde de alta qualidade são essenciais para enfrentar as desigualdades atuais enfrentadas pelas equipes de pesquisa em saúde em ambientes de recursos limitados.<br><br>Este questionário ajudará a identificar qual é a situação atual, lacunas e requisitos para fortalecer a capacidade em ciência de dados em saúde em toda a Ásia, África, América Latina e Caribe, bem como, quais infraestruturas de dados e estruturas de compartilhamento existem em cada região. Ajudará a compreender como o compartilhamento de conhecimento entre instituições, países e regiões é importante para possibilitar um	**Cuestionario - Habilidades de Ciencia de Datos de Salud, Infraestructura de Datos e Intercambio de Datos**<br><br>“La ciencia de datos en la salud es un campo interdisciplinario que utiliza datos, metodología y herramientas para mejorar la salud global. Se beneficia de las matemáticas, estadística, epidemiología e informática para avanzar en la investigación de salud y sus resultados. La ciencia de datos en salud nos ayuda a comprender mejor las enfermedades y las condiciones de salud" (London School of Hygiene and Tropical Medicine).<br><br>El desarrollo de nuevas competencias en ciencia de datos de salud y las habilidades para superar las barreras bien reconocidas que limitan la recopilación, el intercambio, análisis y uso de datos de salud de alta calidad son esenciales para abordar las desigualdades actuales que enfrentan los equipos de investigación en salud en entornos con recursos limitados.<br><br>Este cuestionario ayudará a identificar cuál es la situación actual, las brechas y los requisitos para fortalecer la capacidad en ciencia de datos de salud en Asia, África, América Latina y el Caribe, así como qué infraestructuras de datos y estructuras de intercambio existen en cada región.

countries and regions is important to enabling diverse and large-scale data sharing in a trustworthy way, which can be pivotal to generating insights which improve health policy and practice and improved health outcomes for everyone.	compartilhamento de dados diversificado e em larga escala de forma confiável, o que pode ser crucial para gerar conhecimento que melhore a política e a prática de saúde, assim como a saúde para todos.	Ayudará a comprender la importancia del intercambio de conocimientos entre instituciones, países y regiones para permitir un intercambio de datos fiable y diverso a gran escala, que puede ser crucial para generar conocimientos que mejoren las políticas y prácticas sanitarias, así como los resultados sanitarios para todos.
**Who are we seeking to reach with this survey?**<br>Anyone working in global health data science in Africa, Asia, Latin America and the Caribbean.<br><br>**About the survey**<br>**This three-part survey will take approximately 15 minutes to complete. We greatly appreciate your time in completing it. The survey seeks to understand:**<br><br>• What are the essential health data science skills required, what health data science resources/tools exist in your region, and what are the skills gaps and barriers to skills development?<br>• What health data infrastructure and data sharing platforms, networks, governance frameworks and platforms exist in your region and what types of data you collect and use.?	**A quem estamos buscando alcançar com este questionário?**<br>Qualquer pessoa que trabalhe na área de ciência de dados em saúde global na África, Ásia, América Latina e Caribe.<br><br>**Sobre este questionário**<br>**Este questionário está dividido em três partes, e levará aproximadamente 15 minutos para ser concluído. Agradecemos imensamente o seu tempo ao preenchê-lo. O questionário busca compreender:**<br><br>• Quais são as habilidades essenciais em ciência de dados em saúde, quais recursos/ferramentas de ciência de dados em saúde existem em sua região e quais são as lacunas de habilidades e as barreiras para o desenvolvimento dessas habilidades?<br>• Quais são as infraestruturas de dados em saúde, plataformas de compartilhamento de dados, redes, estruturas de governança e plataformas existentes em sua região e quais tipos de dados você coleta e utiliza?	**¿A quién pretendemos llegar con este cuestionario?**<br>Cualquier persona que trabaje en el campo de la ciencia de datos de salud global en África, Asia, América Latina y el Caribe.<br><br>**Acerca de este cuestionario**<br>**Este cuestionario está dividido en tres partes y tardará aproximadamente 15 minutos en completarse. Agradecemos mucho su tiempo para completarlo. El cuestionario busca comprender:**<br><br>• ¿Cuáles son las habilidades esenciales de ciencia de datos de salud, qué recursos/herramientas de ciencia de datos de salud existen en su región y cuáles son las brechas de habilidades y las barreras para desarrollar esas habilidades?<br>• ¿Cuáles son las infraestructuras de datos de salud, plataformas de intercambio de datos, las redes, estructuras de gobernanza y plataformas existentes en su región, y qué tipos de datos recopila y utiliza?
**Section 1**<br>**Personal Information**<br>**Consent to proceed**	**Seção 1**<br>**Informação pessoal**<br>**Consentimento para prosseguir**	**Sección 1**<br>**Información personal**<br>**Consentimiento para proceder**

Please confirm whether you wish to proceed and complete this survey.<br><br>All data collected via this survey will be stored according to our privacy policy. Your identity will remain anonymous in all publications, release of the data and presentations of the findings. Your identity will only be requested if you give permission for us to keep in contact about this study. Your identity and contact details will not be shared beyond the research team working on this study (members of the team are from Fiocruz, Africa CDC, icddr,b, The Global Health Network and HDR Global). For full details of HDR UK's Privacy Policy please visit https://www.hdruk.ac.uk/privacy-policy/.<br><br>Yes, I agree to processing of the data I have provided and wish to proceed and complete this survey.<br><br>2 Which of the following best describes your primary occupation?<br><br>• Academic (Teacher in Higher Education Institution)<br>• Community Health Worker<br>• Data Analyst<br>• Doctor<br>• Early Career Researcher<br>• Epidemiologist<br>• Laboratory Professional (Manager, Technician etc)<br>• Mathematician<br>• Nurse<br>• Pharmacist	Por favor, confirme se deseja prosseguir e completar este questionário.<br><br>Todos os dados coletados por meio deste questionário serão armazenados de acordo com a nossa política de privacidade. Sua identidade permanecerá anônima em todas as publicações, divulgações dos dados e apresentações dos resultados. Sua identidade será solicitada apenas se você der permissão para entrarmos em contato sobre este estudo. Suas informações pessoais e detalhes de contato não serão compartilhados além da equipe de pesquisa que trabalha neste estudo (membros da equipe são compostos por Fiocruz, Africa CDC, icddr,b, The Global Health Network e HDR Global). Para maiores detalhes sobre a política de privacidade do HDR UK's, por favor visite https://www.hdruk.ac.uk/privacy-policy/.<br><br>Sim, concordo com o processamento dos dados que forneci e desejo prosseguir e concluir este questionário.<br><br>2 Qual das seguintes opções melhor descreve sua ocupação principal?<br><br>• Agente Comunitário de Saúde<br>• Analista de dados<br>• Enfermeiro<br>• Epidemiologista<br>• Estatístico<br>• Estudante<br>• Farmacêutico<br>• Financiador de Pesquisa/Concessor de bolsas<br>• Formulador ou gestor de políticas públicas<br>• Gerente de Projeto/Coordenador de Estudo<br>• Matemático<br>• Médico<br>• Pesquisador Júnior<br>• Pesquisador Sênior/Investigador Principal	Por favor, confirme si desea seguir adelante y completar este cuestionario.<br><br>Todos los datos recopilados a través de este cuestionario se almacenarán de acuerdo con nuestra política de privacidad. Su identidad permanecerá en el anonimato en todas las publicaciones, divulgaciones de datos y presentaciones de resultados. Solo se solicitará su identidad si nos da permiso para ponernos en contacto con usted en relación con este estudio. Su información personal y datos de contacto no se compartirán más allá del equipo de investigación que trabaja en este estudio (los miembros del equipo están compuestos por Fiocruz, Africa CDC, icddr,b, The Global Health Network y HDR Global). Para obtener más detalles sobre la política de privacidad de HDR UK, visite https://www.hdruk.ac.uk/privacy-policy/.<br><br>Sí, estoy de acuerdo con el tratamiento de los datos que he proporcionado y deseo continuar y completar este cuestionario.<br><br>2 ¿Cuál de las siguientes opciones describe mejor su ocupación principal?<br><br>• Analista de datos<br>• Doctor<br>• Enfermera<br>• Epidemiólogo<br>• Estadístico<br>• Estudiante<br>• Farmacéutico<br>• Financiador de Investigación/Profesional de Subvenciones<br>• Formulador o gestor de políticas públicas

- Policy Maker
- Project Manager/Study Coordinator
- Regulatory/Ethics Professional
- Research Funder/Grants Professional
- Senior Researcher/Principal Investigator
- Statistician
- Student
- Other*

*If you selected Other, please specify:

3 Which of the following best describes where you work/study primarily?

- Academic Institution (University, College, etc)
- Biotechnology/Life sciences organisation
- Community Health Centre/Facility
- Government Ministry
- Government Scientific Research Institute
- Hospital
- Immunisation Programme
- Intergovernmental Organisation (IGO)
- International Research Organisation
- Journal/Publishing Company
- Non-Governmental Organisation (NGO)
- Pharmaceutical Organisation
- Regulatory Authority
- Research Funding Organisation
- Technology Organisation
- Other*

*If you selected Other, please specify:

- Professor em Instituição de Ensino Superior
- Profissional de laboratório (Gerente, Técnico, etc.)
- Profissional de regulamentação/ética
- Outro*

* Se você selecionou Outro, por favor especifique:

3 Qual das seguintes opções melhor descreve onde você trabalha/estuda principalmente?

- Autoridade Reguladora
- Centro/Unidade de Saúde
- Hospital
- Instituição Acadêmica (Universidade, Faculdade, etc.)
- Instituto Governamental de Pesquisa Científica
- Ministério Governamental
- Organização de Biotecnologia/Ciências da Vida
- Organização de Financiamento de Pesquisa
- Organização de Tecnologia
- Organização Farmacêutica
- Organização Intergovernamental (OI)
- Organização Internacional de Pesquisa
- Organização Não Governamental (ONG)
- Programa de Imunização
- Revista/Empresa Editorial
- Outro*

* Se você selecionou Outro, por favor especifique:

4 Em qual país você trabalha? Se trabalha em mais de um país, por favor selecione onde trabalha principalmente.

United Nations list of countries provided as a drop-down list

- Gerente de Proyectos/Coordinador de Estudios
- Investigador junior (inicio de carrera)
- Investigador Senior/Investigador Principal
- Matemático
- Profesional de Laboratorio (Gerente, Técnico, etc.)
- Profesional de Regulación/Ética
- Profesor en una Institución de Educación Superior
- Trabajador de Salud Comunitario
- Otro*

* Si seleccionó Otro, especifique:

3 ¿Cuál de las siguientes opciones describe mejor dónde trabajas/estudias principalmente?

- Autoridad Reguladora
- Centro/Unidad de Salud Comunitaria
- Hospital
- Institución académica (universidad, colegio, etc.)
- Instituto Gubernamental de Investigaciones Científicas
- Ministerio del Gobierno
- Organización de Biotecnología/Ciencias de la Vida
- Organización de Financiación de la Investigación
- Organización Farmacéutica
- Organización Intergubernamental (OIG)
- Organización Internacional de Investigación
- Organización no gubernamental (ONG)
- Organización Tecnológica
- Programa de Inmunización
- Revista/Editorial

4 Which country do you work in? If you work in more than one country, please select the country in which you work primarily.<br><br>**United Nations list of countries provided as a drop-down list**		• Otro*<br>* Si seleccionó Otro, especifique:<br><br>4 ¿En qué país trabajas? Si trabajas en más de un país, selecciona el país en el que trabajas principalmente.<br><br>**United Nations list of countries provided as a drop-down list**
**Section 2**<br>**Essential health data science skills/competencies**<br><br>We would like to understand what you consider to be the **essential health data science skills/competencies** that you would need to carry out trustworthy and effective health research using data science approaches.<br><br>Please tick the **three skills** which you consider to be most important in relation to each theme**.** Please also tell us about any **other skills** (not listed) which you consider essential in relation to the theme in the text box provided.<br><br>5 - Top 3 most essential **research planning skills**<br><br>• Developing a research protocol and data science approaches to be applied and seeking ethical approval<br>• Defining the skills required in the research team and data science tools needed	**Seção 2**<br>**Competências/habilidades essenciais em ciência de dados em saúde**<br><br>Gostaríamos de entender o que você considera como **competências/habilidades essenciais em ciência de dados em saúde** necessárias para realizar pesquisas na área da saúde confiáveis e eficazes utilizando abordagens de ciência de dados.<br>Por favor, assinale as **três habilidades** que você considera mais importantes em relação a cada tema. Também informe sobre quaisquer **outras habilidades** (não listadas) que você considere essenciais relacionadas ao tema na caixa de texto fornecida.<br>5 – As 3 principais **habilidades** essenciais para o **planejamento de pesquisa**<br><br>• Desenvolvimento de protocolo de pesquisa e abordagens de ciência de dados a serem aplicadas e buscar por aprovação ética<br>• Definição das habilidades necessárias na equipe de pesquisa e ferramentas de ciência de dados necessárias<br>• Obtenção e gerenciamento do financiamento para pesquisas	**Sección 2**<br>**Competencias/habilidades esenciales en la ciencia de datos de de salud**<br><br>Nos gustaría comprender cuáles considera que son **las competencias/habilidades básicas en ciencia de datos en salud** necesarias para realizar investigaciones de salud confiables y efectivas utilizando enfoques de ciencia de datos.<br>Por favor, marque las **tres habilidades** que considere más importantes en relación con cada tema. También háganos saber sobre cualquier **otra habilidad** (no enumerada) que considere esencial relacionada con el tema en el cuadro de texto proporcionado.<br>5 – Las 3 **habilidades** esenciales para la **planificación de la investigación**<br><br>• Desarrollar un protocolo de investigación y enfoques de ciencia de datos para ser aplicados y buscar la aprobación ética<br>• Definir las habilidades requeridas en el equipo de investigación y las herramientas de ciencia de datos requeridas<br>• Obtener y gestionar la financiación de la investigación

• Sourcing and managing funding awards for research<br>• Understanding of research project management and evaluation<br>• Understanding of the ethical considerations of health data research<br>• This theme is not relevant to my role<br>6 - Please tell us about any **other** essential health data science skills related to **Research Planning** that are missing from the list above. Please separate responses with a comma.<br><br>7 - Top 3 most essential **data access and data management skills**<br><br>• Identifying relevant health data sets for research<br>• Knowledge of different health relevant data sources<br>• Accessing health data sets for research<br>• Capturing and collecting data using appropriate techniques and tools<br>• Understanding of ethical considerations in the use of health data for research<br>• Developing a data management plan<br>• Understanding of data and information governance considerations in relation to use of health data for research<br>• Storing and managing data using appropriate techniques and tools<br>• Making datasets more FAIR (Findable, Accessible, Interoperable, Reusable)	• Compreensão de gestão e avaliação de projetos de pesquisa<br>• Compreensão das considerações éticas da pesquisa de dados de saúde<br>• Este tema não é relevante para minha função<br>6 - Por favor, informe sobre quaisquer **outras** habilidades essenciais em ciência de dados em saúde relacionadas ao **Planejamento de Pesquisa** que estejam ausentes na lista acima. Por favor, separe as respostas com uma vírgula.<br><br>7 – As 3 principais **habilidades** essenciais para **acesso e gestão de dados**<br><br>• Identificação de conjuntos de dados relevantes para pesquisa em saúde<br>• Conhecimento de diferentes fontes de dados relevantes para a saúde<br>• Acesso a conjuntos de dados de saúde para pesquisa<br>• Captura e coleta de dados utilizando técnicas e ferramentas apropriadas<br>• Compreensão das considerações éticas no uso de dados de saúde para pesquisa<br>• Elaboração de um plano de gestão de dados<br>• Compreensão das considerações de governança de dados e informações relacionadas ao uso de dados de saúde para pesquisa<br>• Armazenamento e gestão de dados usando técnicas e ferramentas apropriadas<br>• Tornar conjuntos de dados mais FAIR (Encontráveis, Acessíveis, Interoperáveis, Reutilizáveis)<br>• Preparação de dados, incluindo limpeza, padronização e avaliação de qualidade dos dados antes da análise<br>• Este tema não é relevante para minha função	• Comprensión de la gestión y evaluación de proyectos de investigación<br>• Comprender las consideraciones éticas de la investigación de datos de salud<br>• Este tema no es relevante para mi función<br>6 - Por favor, háganos saber sobre cualquier **otra** habilidad esencial de ciencia de datos de salud relacionada con **la planificación de la investigación** que falte en la lista anterior. Separe las respuestas con una coma.<br><br>7 – Las 3 **habilidades** esenciales para **el acceso y la gestión de datos**<br><br>• Identificación de conjuntos de datos relevantes para la investigación en salud<br>• Conocimiento de diferentes fuentes de datos relevantes para la salud<br>• Acceso a conjuntos de datos de salud para la investigación<br>• Captura y recopilación de datos mediante técnicas y herramientas apropiadas<br>• Comprender las consideraciones éticas en el uso de datos de salud para la investigación<br>• Elaboración de un plan de gestión de datos<br>• Comprensión de la gobernanza de datos y las consideraciones de información relacionadas con el uso de datos de salud para la investigación<br>• Almacenamiento y gestión de datos mediante técnicas y herramientas adecuadas<br>• Hacer que los conjuntos de datos sean más FAIR (Localizables, Accesibles, Interoperables, Reutilizables)

• Data preparation including cleaning, standardising and quality assessment of data prior to analysis<br>• This theme is not relevant to my role<br>8 - Please tell us about any **other** essential health data science skills required for effective **data access and data management.** Please separate responses with a comma.<br><br>**9 -** Top 3 most essential **data analysis skills**<br><br>• Developing a data analysis plan<br>• Understanding of different research methodologies<br>• Identifying appropriate statistical methods for research<br>• Analysing data using different tools and techniques<br>• Presenting data<br>• Data visualisation<br>• This theme is not relevant to my role<br>10 - Please tell us about any **other** essential health data science skills required to carry our effective **analysis.** Please separate responses with a comma.<br><br>**11 -** Top 3 most essential skills for **producing outputs and achieving impact**<br><br>• Critical appraisal of a research paper<br>• Scientific writing for journal publications<br>• Developing a publication and dissemination plan	8 - Por favor, informe sobre quaisquer **outras** habilidades essenciais em ciência de dados em saúde relacionadas ao **Acesso e gestão de dados** que estejam ausentes na lista acima. Por favor, separe as respostas com uma vírgula.<br><br>**9 -** As 3 principais **habilidades** essenciais para **análise de dados**<br><br>• Desenvolvimento de um plano de análise de dados<br>• Compreensão de diferentes metodologias de pesquisa<br>• Identificação de métodos estatísticos apropriados para pesquisa<br>• Análise de dados usando diferentes ferramentas e técnicas<br>• Apresentação de dados<br>• Visualização de dados<br>• Este tema não é relevante para minha função<br>10 - Por favor, informe sobre quaisquer **outras** habilidades essenciais em ciência de dados em saúde necessárias para realizar **análises** eficazes. Por favor, separe as respostas com uma vírgula.<br><br>**11 -** As 3 principais **habilidades** essenciais para **produzir resultados e alcançar impacto**<br><br>• Avaliação crítica de um artigo de pesquisa<br>• Escrita científica para publicações em revistas<br>• Desenvolvimento de um plano de publicação e disseminação<br>• Publicação e disseminação de descobertas de pesquisa por meio de uma variedade de mecanismos<br>• Desenvolvimento de diferentes tipos de resultados de pesquisa (por exemplo, resumos de políticas,	• Preparación de datos, incluida la limpieza de datos, estandarización y evaluación de la calidad antes del análisis<br>• Este tema no es relevante para mi función<br>8 - Por favor, háganos saber sobre cualquier **otra** habilidad esencial en la ciencia de datos de salud relacionada con **el acceso y la gestión de datos** que falte en la lista anterior. Separe las respuestas con una coma.<br><br>**9 -** Las 3 **habilidades** esenciales para **el análisis de datos**<br><br>• Desarrollo de un plan de análisis de datos<br>• Comprensión de las diferentes metodologías de investigación<br>• Identificación de métodos estadísticos apropiados para la investigación<br>• Análisis de datos mediante diferentes herramientas y técnicas<br>• Presentación de datos<br>• Visualización de datos<br>• Este tema no es relevante para mi función<br>10 - Por favor, infórmenos sobre cualquier **otra** habilidad esencial de ciencia de datos de salud necesaria para realizar **análisis efectivos**. Separe las respuestas con una coma.<br><br>**11 -** Las 3 **habilidades** esenciales para **producir resultados y lograr impacto**<br><br>• Valoración crítica de un trabajo de investigación<br>• Redacción Científica para publicaciones en revistas

• Publishing and disseminating research findings through a range of mechanisms<br>• Developing different types of research outputs (e.g. policy briefs, apps, tools, dashboards)<br>• Monitoring and evaluating the impact of research through a range of mechanisms<br>• This theme is not relevant to my role<br>12 - Please tell us about any **other** essential health data science skills required for effectively **producing outputs and achieving impact.** Please separate responses with a comma.<br><br>13 - Top 3 most essential skills for **stakeholder engagement** (local communities, health practitioners, policy makers, health research funders)<br><br>• Developing a stakeholder engagement plan<br>• Knowledge and understanding of effective methodologies to engage with communities/stakeholders<br>• Working with different stakeholders to ensure their interests and perspectives are considered<br>• Communicating research evidence to influence health policy and practice<br>• Communicating research at different levels through engaging with a range of stakeholders<br>• This theme is not relevant to my role	aplicativos, ferramentas, painéis de dados interativos)<br>• Monitoramento e avaliação do impacto da pesquisa por meio de uma variedade de mecanismos<br>• Este tema não é relevante para minha função<br>12 - Por favor, informe sobre quaisquer **outras** habilidades essenciais em ciência de dados em saúde necessárias para **produzir resultados de forma eficaz e alcançar impacto**. Por favor, separe as respostas com uma vírgula.<br><br>13 - As 3 principais **habilidades** essenciais para **engajamento de partes interessadas** (comunidades locais, profissionais de saúde, tomadores de decisão em políticas de saúde, financiadores de pesquisa em saúde)<br><br>• Desenvolvimento de um plano de engajamento de partes interessadas<br>• Conhecimento e compreensão de metodologias eficazes para engajar comunidades/partes interessadas<br>• Trabalhar com diferentes partes interessadas para garantir que seus interesses e perspectivas sejam considerados<br>• Comunicar evidências de pesquisa para influenciar políticas e práticas de saúde<br>• Comunicar a pesquisa em diferentes níveis por meio do engajamento com uma variedade de partes interessadas<br>• Este tema não é relevante para minha função<br>14 - Por favor, informe sobre quaisquer **outras** habilidades essenciais em ciência de dados em saúde necessárias para o **engajamento eficaz de partes**	• Elaboración de un plan de publicación y difusión<br>• Publicación y difusión de los resultados de la investigación a través de diversos mecanismos.<br>• Desarrollo de diferentes tipos de resultados de búsqueda (por ejemplo, resúmenes de políticas, aplicaciones, herramientas, tableros de datos interactivos)<br>• Monitorear y evaluar el impacto de la investigación a través de una variedad de mecanismos.<br>• Este tema no es relevante para mi función<br>12 - Por favor, háganos saber sobre cualquier **otra** habilidad esencial de ciencia de datos de salud necesaria para **producir resultados de manera efectiva y lograr impacto**. Separe las respuestas con una coma.<br><br>13 - Las 3 **habilidades** esenciales para la **participación de las partes interesadas** (comunidades locales, profesionales de la salud, responsables de la toma de decisiones en materia de políticas sanitarias, financiadores de la investigación sanitaria)<br><br>• Desarrollo de un plan de participación de las partes interesadas<br>• Conocimiento y comprensión de metodologías efectivas para involucrar a las comunidades/partes interesadas<br>• Trabajar con diferentes partes interesadas para garantizar que se tengan en cuenta sus intereses y perspectivas.

14 - Please tell us about any **other** essential health data science skills required for effective **stakeholder engagement.** Please separate responses with a comma.	**interessadas**. Por favor, separe as respostas com uma vírgula.	• Comunicar la evidencia de la investigación para influir en las políticas y prácticas de salud<br>• Comunicar la investigación a diferentes niveles a través de la participación de una variedad de partes interesadas<br>• Este tema no es relevante para mi función<br>14 - Por favor, infórmenos sobre cualquier **otra** habilidad esencial de ciencia de datos de salud necesaria para **una participación efectiva de las partes interesadas**. Separe las respuestas con una coma.
**Section 3**<br><br>**Health data science resources, skills gaps, barriers to skills development and solutions**<br><br>We are interested in learning about what freely available health data science training courses or resources are available in your region. This will help us to identify existing resources which we can signpost to and build on.<br><br>We would also like to understand any health data science knowledge or skills gaps or practical barriers to carrying out trustworthy and effective health research using data sciences approaches. and how these could be addressed.<br><br>15 - Do you know of any freely available **health data science training resources or courses** in your region?	**Seção 3**<br><br>**Recursos em ciência de dados em saúde, lacunas de habilidades, barreiras para o desenvolvimento de habilidades e soluções**<br><br>Estamos interessados em saber quais cursos ou recursos de treinamento gratuitos em ciência de dados em saúde estão disponíveis em sua região. Isso nos ajudará a identificar recursos existentes aos quais podemos referenciar e ampliar.<br><br>Também gostaríamos de entender quaisquer lacunas de conhecimento ou habilidades em ciência de dados em saúde, ou barreiras práticas para realizar pesquisas confiáveis e eficazes em saúde usando abordagens de ciências de dados, e como essas lacunas poderiam ser superadas.<br><br>15 - Você conhece algum **recurso ou curso de treinamento em ciência de dados em saúde** disponível gratuitamente em sua região?<br><br>• Sim	**Sección 3**<br><br>**Recursos en ciencia de datos de salud, brechas de habilidades, barreras para el desarrollo de habilidades y soluciones**<br><br>Estamos interesados en saber qué cursos o recursos gratuitos de capacitación en ciencia de datos de salud están disponibles en su región. Esto nos ayudará a identificar los recursos existentes a los que podemos remitirnos y ampliarlos.<br><br>También nos gustaría comprender las brechas en el conocimiento o las habilidades en la ciencia de datos de salud, o las barreras prácticas para realizar investigaciones de salud confiables y efectivas utilizando enfoques de ciencia de datos, y cómo se podrían superar estas brechas.<br><br>15 - ¿Conoce algún **recurso de ciencia de datos de salud o cursos de capacitación disponibles de forma** gratuita en su región?<br><br>• Sí

• Yes<br>• No<br>16 - Please tell us about any freely available **health data science training courses or resources** which you are aware of in your region, and which skills they seek to develop.<br><br>(e.g. courses, toolkits or handbooks for R or other skills, 'how to' tutorials)<br><br>17 - Do you know of any **health data science skills or knowledge gaps or barriers to skills development in your region** which prevent you or others from doing more health research using data science approaches?<br><br>• Yes<br>• No<br>18 - Please tell us about **health data science knowledge and skills gaps** which prevent you from doing more health research. Please tell us about the **practical barriers that prevent you and others from doing more health research** or developing your/their own research skills and experience?<br><br>E.g. Lack of specific skills, lack of funding for training, lack of relevant courses, limited access to or knowledge of datasets, challenges in data sharing, lack of infrastructure.<br><br>19 - Do you know of any **solutions which may help to address the skills gaps and barriers to skills development**?<br><br>• Yes	• Não<br>16 - Por favor, informe sobre quaisquer **cursos ou recursos de treinamento em ciência de dados em saúde** disponíveis gratuitamente de que você tem conhecimento em sua região, e que habilidades eles buscam desenvolver.<br><br>(por exemplo, cursos, ferramentas - toolkits ou manuais para R ou outras habilidades, tutoriais 'como fazer')<br><br>17 - Você conhece alguma **lacuna de conhecimento ou barreiras para o desenvolvimento de habilidades** em ciência de dados em saúde em sua região, que impeçam você ou outros de realizar mais pesquisas em saúde usando abordagens de ciência de dados?<br><br>• Sim<br>• Não<br>18 - Por favor, informe sobre l**acunas de conhecimento e habilidades em ciência de dados em saúde** que impedem você de realizar mais pesquisas em saúde. Por favor, comente sobre as **barreiras práticas que impedem você e outros de realizar mais pesquisas em saúde** ou desenvolver suas próprias habilidades e experiência de pesquisa?<br><br>Ex.: falta de habilidades específicas, falta de financiamento para treinamento, ausência de cursos relevantes, acesso limitado ou falta de conhecimento sobre conjuntos de dados, desafios no compartilhamento de dados, falta de infraestrutura.<br><br>19 - Você conhece alguma **solução que possa ajudar a lidar com as lacunas de habilidades e as barreiras para o desenvolvimento de habilidades**?<br><br>• Sim	• No<br>16 - Por favor, háganos saber sobre cualquier **curso o recurso de capacitación en ciencia de datos de salud** disponible gratuitamente que conozca en su región, y qué habilidades buscan desarrollar.<br><br>(por ejemplo, cursos, herramientas - toolkits o manuales para R u otras habilidades, tutoriales prácticos)<br><br>17 - ¿Conoce alguna **brecha de conocimiento o barrera para el desarrollo** de habilidades de ciencia de datos de salud en su región, que le impida a usted u otros realizar más investigaciones de salud utilizando enfoques de ciencia de datos?<br><br>• Sí<br>• No<br>18 - Por favor, háganos saber sobre las áreas **de conocimiento y habilidades en la ciencia de datos de salud** que le impiden realizar más investigaciones en salud. Por favor, cuéntenos sobre **las barreras prácticas que le impiden a usted y a otros realizar más investigaciones sobre la salud** o desarrollar sus propias habilidades y experiencia en investigación.<br><br>Por ejemplo: falta de habilidades específicas, falta de financiación para la formación, ausencia de cursos pertinentes, acceso limitado o falta de conocimiento sobre conjuntos de datos, dificultades para compartir datos, falta de infraestructura.

• No<br>20 - Please tell us **how you think the skills gaps/barriers can be addressed.**	• Não<br>20 - Por favor, comente sobre **como você acha que as lacunas/barreiras de habilidades podem ser abordadas.**	19 - ¿Conoces alguna **solución que pueda ayudar a abordar las brechas de habilidades y las barreras para el desarrollo de habilidades**?<br>• Sí<br>• No<br>20 - Por favor, díganos **cómo cree que se pueden abordar las carencias/barreras de habilidades.**
**Section 4**<br>**Thank you**<br>Thank you for participating in this survey. We greatly appreciate your time and insights. The information you have provided will be used to shape priorities moving forward to best enable health researchers and health practitioners in your region to use health data science approaches to improve health outcomes for everyone, and to inform policy makers and funders of the gaps and barriers to using health data science approaches. Please let us know below if you would like to receive an update on the findings of this survey.<br>21 - Would you like to receive an update on findings of this survey?<br>• Yes<br>• No	**Seção 4**<br>**Obrigado**<br>Obrigado por responder este questionário. Agradecemos imensamente o seu tempo e contribuições. As informações fornecidas serão utilizadas para definir prioridades visando capacitar da melhor forma possível os pesquisadores e profissionais de saúde em sua região a utilizar abordagens de ciência de dados em saúde para melhorar os resultados de saúde para todos, além de informar os formuladores de políticas e financiadores sobre as lacunas e obstáculos no uso dessas abordagens. Por favor, informe abaixo se gostaria de receber uma atualização sobre os resultados deste questionário.<br>21 - Você gostaria de receber atualizações sobre os resultados deste questionário?<br>• Sim<br>• Não	**Sección 4**<br>**Gracias**<br>Gracias por responder a este cuestionario. Agradecemos mucho su tiempo y contribuciones. La información proporcionada se utilizará para establecer prioridades con el fin de empoderar mejor a los investigadores y profesionales de la salud de su región para que utilicen los enfoques de la ciencia de datos sanitarios para mejorar los resultados de salud para todos, así como para informar a los formuladores de políticas y a los financiadores sobre las lagunas y los obstáculos en el uso de estos enfoques. Háganos saber a continuación si desea recibir una actualización sobre los resultados de este cuestionario.<br>21 - ¿Le gustaría recibir actualizaciones sobre los resultados de este cuestionario?<br>• Sí<br>• No
**Section 5**<br>**Details**<br>22 - Please provide your **name** for communications	**Seção 5**<br>**Detalhes**<br>22 – Por favor informe seu **nome** para receber informações	**Sección 5**<br>**Detalles**<br>22 – Por favor, proporcione su **nombre** para recibir información

23 - Please provide your **email address** for communications	23 – Por favor informe seu **e-mail** para receber informações	23 – Por favor, informe su **correo electrónico** para recibir información
