# Supplementary material for: Exploring the landscape of essential health data science skills and research challenges: a survey of stakeholders in Africa, Asia, and Latin America and the Caribbean
Source: Front Public Health. 2025 Mar 28;13:1523873. doi: 10.3389/fpubh.2025.1523873 (PMC11985845; doi:10.3389/fpubh.2025.1523873)
Supplement: Supplementary file 6 [file Data_Sheet_3.PDF]

Supplementary material 4 – Appendix 1 - List of data science skills used in the essential data science skills landscaping survey

Research planning

- Developing a research protocol and data science approaches to be applied and seeking ethical approval
- Defining the skills required in the research team and data science tools needed
- Sourcing and managing funding awards for research
- Understanding of research project management and evaluation
- Understanding of the ethical considerations of health data research

Data access and data management

- Identifying relevant health data sets for research
- Knowledge of different health relevant data sources
- Accessing health data sets for research
- Capturing and collecting data using appropriate techniques and tools
- Understanding of ethical considerations in the use of health data for research
- Developing a data management plan
- Understanding of data and information governance considerations in relation to use of health data for research
- Storing and managing data using appropriate techniques and tools
- Making datasets more FAIR (Findable, Accessible, Interoperable, Reusable)
- Data preparation including cleaning, standardising and quality assessment of data prior to analysis

Data analysis

- Developing a data analysis plan
- Understanding of different research methodologies
- Identifying appropriate statistical methods for research
- Analysing data using different tools and techniques
- Presenting data
- Data visualisation

Producing outputs and achieving impact

- Critical appraisal of a research paper
- Scientific writing for journal publications
- Developing a publication and dissemination plan
- Publishing and disseminating research findings through a range of mechanisms
- Developing different types of research outputs (e.g. policy briefs, apps, tools, dashboards)
- Monitoring and evaluating the impact of research through a range of mechanisms

Stakeholder engagement (local communities, health practitioners, policy makers, health research funders)

- Developing a stakeholder engagement plan
- Knowledge and understanding of effective methodologies to engage with communities/stakeholders
- Working with different stakeholders to ensure their interests and perspectives are considered
- Communicating research evidence to influence health policy and practice
- Communicating research at different levels through engaging with a range of stakeholders
